# Yeast Smy2 and its human homologs GIGYF1 and -2 regulate Cdc48/VCP function during transcription stress

**DOI:** 10.1016/j.celrep.2022.111536

**Published:** 2022-10-25

**Authors:** Michelle Harreman Lehner, Jane Walker, Kotryna Temcinaite, Anna Herlihy, Michael Taschner, Adam C. Berger, Anita H. Corbett, A. Barbara Dirac Svejstrup, Jesper Q. Svejstrup

**Affiliations:** 1 Mechanisms of Transcription Laboratory, The Francis Crick Institute, 1 Midland Road, London NW1 1AT, UK; 2 Department of Biology, RRC 1021, Emory University, 1510 Clifton Road, NE, Atlanta 30322, GA, USA; 3 Department of Cellular and Molecular Medicine, Panum Institute, University of Copenhagen, Blegdamsvej 3B, 2200 Copenhagen N, Denmark

**Keywords:** SMY2, GIGYF1, GIGYF2, cdc48, p97, VCP, RNA polymerase II, last resort pathway, DEF1, proteasome

## Abstract

The “last resort” pathway results in ubiquitylation and degradation of RNA polymerase II in response to transcription stress and is governed by factors such as Def1 in yeast. Here, we show that the *SMY2* gene acts as a multi-copy suppressor of *DEF1* deletion and functions at multiple steps of the last resort pathway. We also provide genetic and biochemical evidence from disparate cellular processes that Smy2 works more broadly as a hitherto overlooked regulator of Cdc48 function. Similarly, the Smy2 homologs GIGYF1 and -2 affect the transcription stress response in human cells and regulate the function of the Cdc48 homolog VCP/p97, presently being explored as a target for cancer therapy. Indeed, we show that the apoptosis-inducing effect of VCP inhibitors NMS-873 and CB-5083 is *GIGYF1/2* dependent.

## Highlights

• Smy2 and its human homologs GIGYF1/2 function in the transcription stress response
• These proteins regulate the function of CDC48/VCP, also in other cellular processes
• Deletion of *GIGYF1/2* renders cells less sensitive to VCP inhibition

## Graphical abstract

**Figure:**
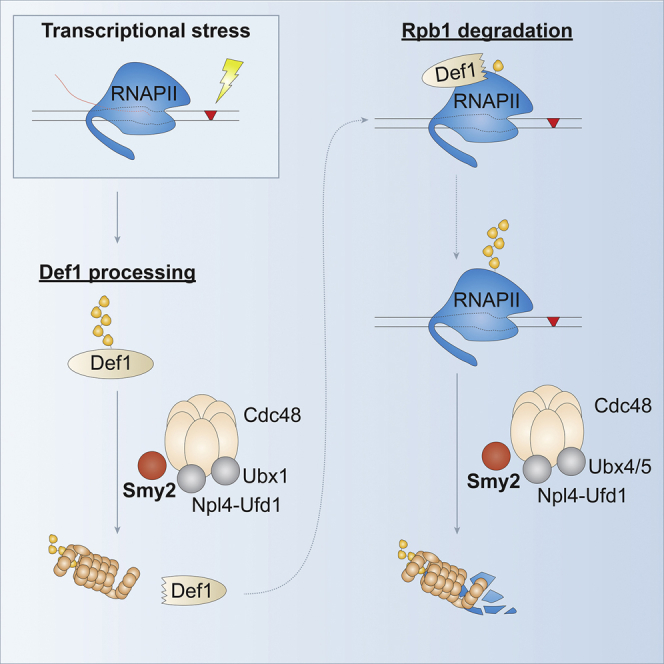

## Teaser

Lehner et al. describe the function of the yeast Smy2 protein in the transcription stress response, where it affects extraction of ubiquitylated RNA polymerase II from chromatin. Smy2, and its human homologs GIGYF 1/2, regulate the function of the protein segregase CDC48/VCP in transcription but also in other cellular processes.

## Introduction

The “last resort” pathway is induced by cellular stresses such as UV-induced DNA damage and involves multiple components of the ubiquitin-proteasome system (UPS) (Wilson et al., 2013a). The budding yeast Def1 protein plays a key role in this pathway, which ultimately results in ubiquitylation and degradation of stalled or arrested RNA polymerase II (RNAPII), for example at transcription-blocking DNA lesions and in response to other transcription problems (Wilson et al., 2013a; Noe Gonzalez et al., 2021). Indeed, Def1 allows the recruitment of the Elc1-Ela1-Cul3 ligase complex to damage stalled/arrested RNAPII (Wilson et al., 2013b), which is then poly-ubiquitylated on the Rpb1 subunit and extracted from chromatin by Cdc48 and its co-factors Ubx4 and -5 (Verma et al., 2011) to facilitate degradation by the proteasome (Wilson et al., 2013a; Noe Gonzalez et al., 2021). Interestingly, activation of the response initially entails protein processing (but not degradation) of Def1 by the proteasome (Wilson et al., 2013b), an activation process by protein “clipping” that has also been observed with transcription proteins such as Spt23 and Mga2 in other contexts and for which the activity of Cdc48 is also required (Hoppe et al., 2000; Rape et al., 2001; Shcherbik and Haines, 2007; Kolawa et al., 2013).

Cdc48 (also known as VCP/p97 in mammals, Ter94 in *Drosophila melanogaster*, and CDC-48 in *Caenorhabditis elegans*) is a hexameric protein of the ATPases associated with diverse cellular activities (AAA) family. This protein “segregase” uses the energy of ATP hydrolysis to structurally remodel a multitude of target proteins (Erzberger and Berger, 2006; Ye et al., 2017). The function of Cdc48/VCP is typically ubiquitin dependent, with CDC48/VCP binding to ubiquitylated substrates and facilitating UPS steps downstream of ubiquitylation (Stach and Freemont, 2017). For example, Cdc48/VCP extracts ubiquitylated proteins from disparate cellular structures ranging from membranes to chromatin or segregates them from their associated partner proteins, expediating the subsequent degradation by the proteasome (van den Boom and Meyer, 2018). Cdc48/VCP may also enable the creation of flexible proteasome initiation regions in protein substrates that might otherwise be refractory to degradation (Olszewski et al., 2019).

Cdc48/VCP associates with scores of co-factors, required for the correct regulation of its involvement in different cellular processes (Buchberger et al., 2015; Hanzelmann and Schindelin, 2017). These co-factors, which include proteins such as the Ufd1-Npl4 dimer and the Ubx adaptor proteins, are defined by specific interaction domains or motifs that allow their association with Cdc48/VCP and ubiquitin (Schuberth and Buchberger, 2008; Meyer et al., 2012). Some also serve as ubiquitin adaptors or recruit the segregase to act at specific subcellular structures. The association of Cdc48/VCP with co-factors is often exceptionally dynamic (Xue et al., 2016), opening the possibility that additional regulatory co-factors remain to be discovered.

Here, we describe experiments that lead us to propose that yeast Smy2 and its human homologs GIGYF1 and -2 (GIGYF1/2) play an important role in the transcription stress response, most likely as previously overlooked, general regulators of Cdc48/VCP function.

## Results

### *SMY2* is a multi-copy suppressor of *def1Δ*

**Figure 1:**
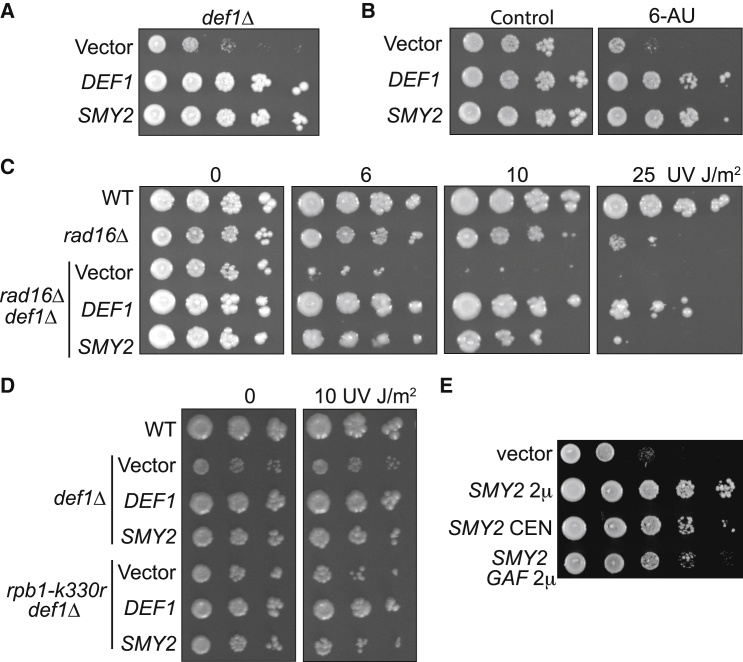
Over-expression of *SMY2* suppresses *def1Δ* phenotypes (A) Dilution series on yeast minimal media plates of *def1Δ* cells carrying multi-copy (2 μm) plasmids expressing *SMY2* or a *CEN* plasmid expressing *DEF1*. (B) As in (A) but in the presence of 6-AU. (C) As in (A) but effect on the growth of *def1Δ* and *def1Δ rad16Δ* cells, in the presence or absence of UV irradiation as indicated. (D) As in (C) but in *def1Δ rpb k330r* cells. (E) As in (A) but after mutation of the Smy2 GYF motif (*SMY2 GAF*).

Yeast cells lacking the last resort protein *DEF1* gene (*def1Δ*) are slow growing and defective in RNAPII poly-ubiquitylation and degradation (Woudstra et al., 2002). We exploited the sensitivity of *def1Δ* cells to benomyl (Parsons et al., 2004) to identify suppressors. Using a library of multi-copy (2 μm) plasmids expressing yeast genes (Zhang et al., 1998), only two distinct suppressors of slow growth were identified. Not surprisingly, one of these was *DEF1* itself. The second identified suppressor was suppressor of myo2-66 (*SMY2*) (Lillie and Brown, 1994). *SMY2* over-expression rescued the slow-growth phenotype of *def1Δ* to an extent akin to that achieved by expression of *DEF1* itself (Figure 1A).

To investigate whether *SMY2* can suppress *def1Δ* phenotypes associated with the last resort pathway for ubiquitylation and degradation of RNAPII (Wilson et al., 2013a), we first tested growth inhibition in the presence of the elongation inhibitor 6-azauracil (6-AU), which causes transcription stress at least partly by depleting the intracellular nucleotide pool (Riles et al., 2004; Mason and Struhl, 2005). *def1Δ* cells grow slowly on media containing 6-AU (Woudstra et al., 2002), which can be rescued by *SMY2* over-expression (Figure 1B).

Although *def1Δ* cells are not themselves sensitive to UV irradiation, loss of *DEF1* substantially increases the UV sensitivity of cells lacking the *RAD16* nucleotide excision repair (NER) gene (Woudstra et al., 2002) (compare Figure 1C, vector control, with *rad16Δ* after UV irradiation). *SMY2* over-expression markedly suppressed the elevated UV sensitivity of *def1Δ rad16Δ* cells (see, for example, 10 J/m^2^), further linking it to the last resort pathway.

When combined with a mutation that affects ubiquitylation of Rpb1 (*rpb1*-*k330r*; Somesh et al., 2007), *def1Δ* cells are also slightly UV sensitive (Figure 1D). Interestingly, while *SMY2* over-expression rescued the slow growth of *def1Δ rpb1-k330r*, it failed to rescue its UV sensitivity. This observation suggests that ubiquitylation of Rpb1 is required to facilitate the rescue function of Smy2 after DNA damage, pointing to a possible connection between Smy2 and the UPS.

Smy2 is an 81 kDa protein with a glycine-tyrosine-phenylalanine (GYF) motif (Kofler and Freund, 2006). To determine if the GYF motif plays a role in *SMY2*-mediated suppression of *def1Δ*, we generated a mutant of Smy2 where tyrosine_234_ is changed to an alanine (GYF→GAF; *smy2 gaf*). This mutation has previously been shown to compromise the function of Smy2 (Sezen et al., 2009). High-copy expression of *smy2 gaf* yielded levels of mutated Smy2 protein that were similar to wild type (WT) (data not shown) but only partially suppressed the *def1Δ* slow-growth phenotype (Figure 1E).

### Smy2 functions with Cdc48

**Figure 2:**
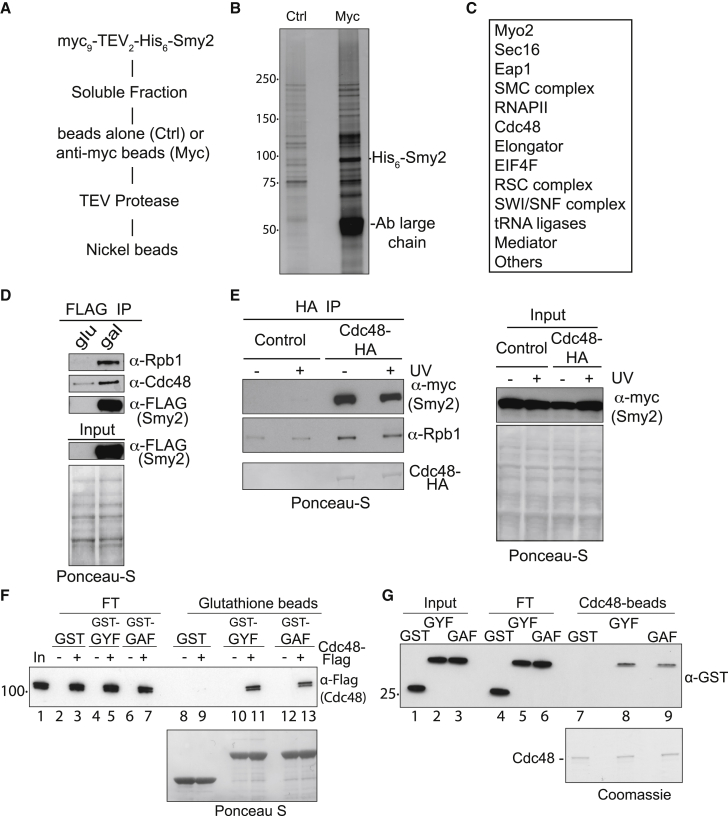
Smy2 interacts with Cdc48 (A) Smy2 purification scheme for mass spectrometry analysis. (B) Silver staining of proteins co-purifying with Myc-tagged Smy2. (C) Examples of proteins and protein complexes (several subunits detected) co-purifying with Smy2. Data for all interactors are found in Table S1. (D) FLAG coIP of Rpb1 and Cdc48 in cells expressing FLAG-tagged Smy2 from the *GAL* promoter (gal) or not (glu). (E) Left, HA coIP of Smy2 and Rpb1 in cells expressing Cdc48-HA_3_ (or not [control]). The Cdc48-HA_3_ from the beads is detected by Ponceau S staining. Right, inputs. (F) Western blot showing interaction between purified Cdc48 protein and glutathione beads bound with recombinant GST, GST-tagged Smy2 GYF, or the mutated version of the domain (GAF). Ponceau S staining was used to visualize protein on the beads. (G) As in (C) but the reverse interaction between purified, immobilized Cdc48 and GST-GYF proteins. Coomassie staining shows Cdc48 from beads. See also Table S1 and Figure S1.

The yeast genetic experiments above suggested a role for Smy2 in the cellular response to transcription stress, which is particularly evident in *def1Δ* cells, but how this occurs was unclear. To better understand the role of Smy2, we genetically engineered the *SMY2* gene to add epitope tags to either the N or C terminus of Smy2. C-terminal-tagged Smy2 failed to rescue *def1* deletion, suggesting that such tagging negatively affects Smy2 function, but Smy2 with N-terminal tags was invariably functional (data not shown). We used a yeast strain expressing Myc_9_-TEV_2_-His_6_-Smy2 to perform mass spectrometric analysis of Smy2 interactors, via a two-step purification protocol (Figure 2A). After purification, several protein bands were apparent that were absent with the untagged control (Figure 2B). The eluates were subjected to mass spectrometry analysis, which identified numerous Smy2 interactors; among these were Myo2, Sec16, and Eap1, which have previously been shown to genetically and/or physically interact with Smy2 (Lillie and Brown, 1994; Higashio et al., 2008; Sezen et al., 2009). Interestingly, RNAPII and Cdc48 were also identified as Smy2 interactors (a partial list of interactors is shown in Figure 2C; a complete list with additional data is shown in Table S1).

Cdc48 is essential during transcription stress as a key UPS component of the last resort pathway; here, its function is to extrude poly-ubiquitylated Rpb1 from RNAPII in chromatin for efficient proteasomal degradation (Verma et al., 2013; Lafon et al., 2015). To further analyze the intriguing connection between Smy2, Rpb1, and Cdc48, we performed immunoprecipitation experiments from a yeast strain expressing galactose-inducible FLAG_3_-Smy2 (Figure 2D). In our hands, Cdc48 is a “sticky” protein that may bind non-specifically to proteins and resins, but immunoblotting revealed that Cdc48 and Rpb1 were only markedly co-immunoprecipitated (coIPed) when FLAG_3_-Smy2 was expressed (Figure 2D, gal). As a further confirmation of these interactions, we also used anti-hemagglutinin (HA) antibody to IP from extracts of congenic Myc-tagged Smy2 strains, only one of which expressed HA-tagged Cdc48 (Figure 2E). Smy2 was specifically coIPed with tagged Cdc48, independently of UV irradiation. Cdc48 also co-precipitated Rpb1 independently of UV irradiation, in agreement with previous work (Verma et al., 2011).

As Cdc48 and Smy2 might directly interact, and Smy2 contains a GYF motif, we also tested if a Smy2 fragment containing this motif (Smy_205-261_) might help facilitate the interaction. We also further changed the GYF domain (GYF→GAA) in the hope of generating a more penetrant *smy2* mutant, but this resulted in the protein fragment becoming insoluble (data not shown). Epitope-tagged versions of Cdc48 were purified from yeast extracts, while recombinant GST and GST-Smy_205-261_ were purified to near homogeneity after bacterial expression. This Smy2 fragment bound to purified Cdc48, and the binding observed with the mutated (GAF) version was only slightly reduced, both when tested with an immobilized Smy2 fragment (Figure 2F, compare lanes 11 and 13, also with lane 9) and with purified, immobilized Cdc48 (Figure 2G, compare lanes 8 and 9, also with lane 7).

**Figure 3:**
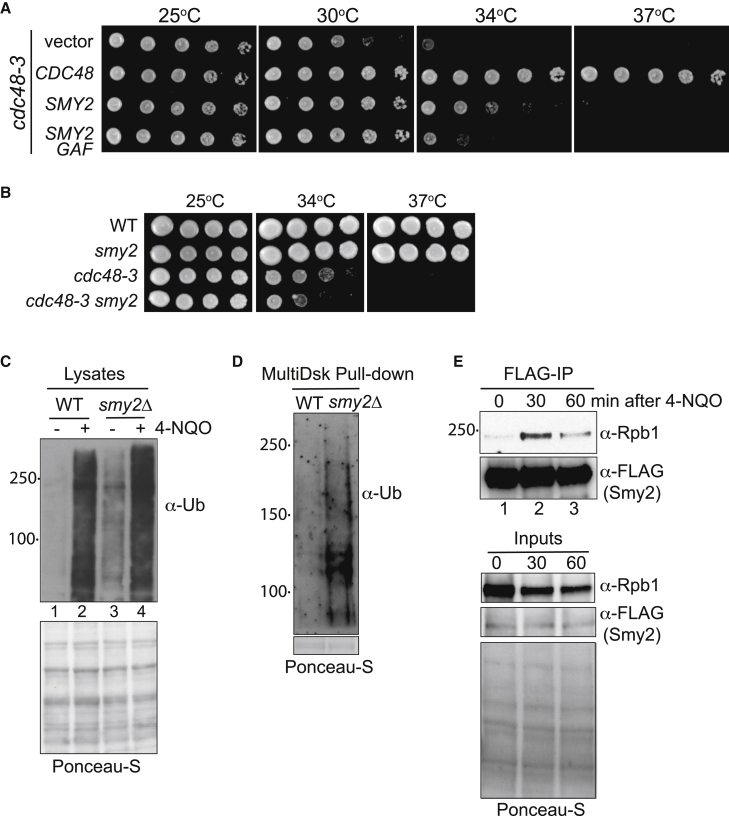
Smy2 works with Cdc48 and affects protein ubiquitylation (A) Dilution series of *cdc48-3* cells (carrying the indicated 2-μm plasmids) plated on minimal media and grown at the specified temperatures for 2–4 days. (B) Dilution series as in (A), showing the effect of *cdc48* mutation, deletion of *SMY2*, or both. (C) Western blot of ubiquitylated proteins from wild-type (WT) and *smy2Δ* cells before or after DNA damage with 4-NQO. (D) As in (A) but ubiquitylated proteins isolated by MultiDsk affinity chromatography. Here, a 3%–8% gel was run until the 75-kDa band had reached the bottom of the gel. The Ponceau S stain (bottom panel) shows the eluted GST-MultiDsk protein. (E) Top, western blot showing coIP of RNAPII (Rpb1) with FLAG-tagged Smy2 at different times after DNA damage. Bottom, inputs. Note that it was not possible in these experiments to detect poly-ubiquitylated Rpb1 due to the weak signal.

The biochemical experiments above indicate that Cdc48 and Smy2 interact. To investigate whether and how Smy2 and Cdc48 function might be related, we tested if high-copy expression of *SMY2* can suppress the slow growth phenotype of the temperature-sensitive *cdc48-3* strain (Figure 3A). *cdc48-3* cells grow like WT at 25°C but are slow growing at 30°C and show no significant growth at 34°C and 37°C (Latterich et al., 1995). As expected, expression of WT *CDC48* rescued *cdc48-3* cells at all tested temperatures. Remarkably, however, over-expression of *SMY2* rescued the slow growth of *cdc48-3* cells at 30°C and partially at 34°C. Furthermore, *smy2 gaf* suppressed *cdc48-3* cells at 30°C but less than WT *SMY2* at 34°C, suggesting a partial dependence on the GYF domain in *cdc48-3* suppression, as observed above for *def1Δ* suppression.

These data are consistent with previous high-throughput genetic screens, which isolated *SMY2* as one of several candidate suppressors of *cdc48-3’*s temperature sensitivity (Magtanong et al., 2011). This prior work failed to identify Smy2 as a suppressor of two other Cdc48 mutants (*cdc48-2* and *cdc48-9*). However, given that high-throughput screens are frequently not exhaustive, we re-tested whether *SMY2* over-expression can in fact also alleviate the temperature sensitivity of these *cdc48* strains. Indeed, *SMY2* expression suppressed both *cdc48-9* and *cdc48-2* as well, while the *SMY2 GYF→GAF* mutation again only partially negated suppression (Figure S1). These data indicate that over-expression of Smy2 can compensate for defects in Cdc48 function, providing strong genetic evidence for a functional relationship. To determine if *SMY2* and *CDC48* have a synthetic genetic interaction, we examined the effect of deleting *SMY2* in *cdc48-3* cells. This resulted in exacerbated temperature sensitivity (Figure 3B), indicating synthetic interaction.

Together, these data provide genetic support for the idea that Smy2 works with Cdc48.

### Evidence that Smy2 regulates Cdc48 function

The data above support the idea that Smy2 and Cdc48 physically and genetically interact and raised the possibility that this may be important during transcription stress. Cdc48 has numerous cellular targets and is coupled to many different adaptors and regulators to orchestrate the cellular response to protein ubiquitylation (Stach and Freemont, 2017; van den Boom and Meyer, 2018). For example, Ubx4 and Ubx5, from the Ubx family of Cdc48 adaptors, are required for Cdc48-mediated extrusion of stalled poly-ubiquitylated Rpb1 upon DNA damage (Verma et al., 2011). Considering these data in light of our own, we hypothesized that Smy2 might be a previously unrecognized type of regulator of Cdc48 function. Strains carrying mutations in *CDC48* or its adaptor/regulator proteins accumulate ubiquitin conjugates (Kolawa et al., 2013), in all likelihood due to target proteins not being efficiently extruded from partners or cellular structures for proteasome-mediated degradation. To determine if Smy2 deficiency also elicits a global effect on protein ubiquitylation, we compared the global level of ubiquitylated proteins in *smy2Δ* cells with that in WT. Immunoblotting for ubiquitin indeed revealed an accumulation of ubiquitin conjugates in *smy2Δ* cells (Figure 3C, compare lanes 1 and 3), which was exacerbated by incubation with the UV-damage mimetic 4-nitroquinoline N-oxide (4-NQO) (Figure 3C, compare lanes 3 and 4 and lanes 2 and 4). We also exploited MultiDsk beads (Wilson et al., 2012) to isolate poly-ubiquitylated proteins (Anindya et al., 2007; Tufegdzic Vidakovic et al., 2019). Immunoblotting for ubiquitin after such enrichment again showed a clear and consistent accumulation of ubiquitin species in *smy2Δ* cells (Figure 3D). These data indicate that Smy2 affects cellular ubiquitylation levels.

To investigate possible functions for Smy2 during transcription stress-inducing DNA damage, we probed its binding to Rpb1. Cells expressing FLAG-Smy2 were exposed to 4-NQO and harvested at different time points. FLAG IP from cellular lysates was then performed before immunoblotting for FLAG-Smy2 and Rpb1. To detect Rpb1, we used the 4H8 antibody, which recognizes most phosphorylated forms of Rpb1. This is helpful, as the unphosphorylated and phosphorylated forms of Rpb1 in yeast are difficult to distinguish by gel mobility. As expected after DNA damage, the elongating, phosphorylated form of Rpb1 was somewhat depleted over the time course of the experiment (Figure 3E, inputs). However, although there was less total Rpb1 in the input lysate after 4-NQO treatment, there was more Rpb1 co-immunoprecipitating with FLAG-Smy2, especially 30 min after 4-NQO treatment (Figure 3E, compare lanes 1 and 2), indicating an increase in Smy2 interaction with phosphorylated RNAPII after DNA damage.

**Figure 4:**
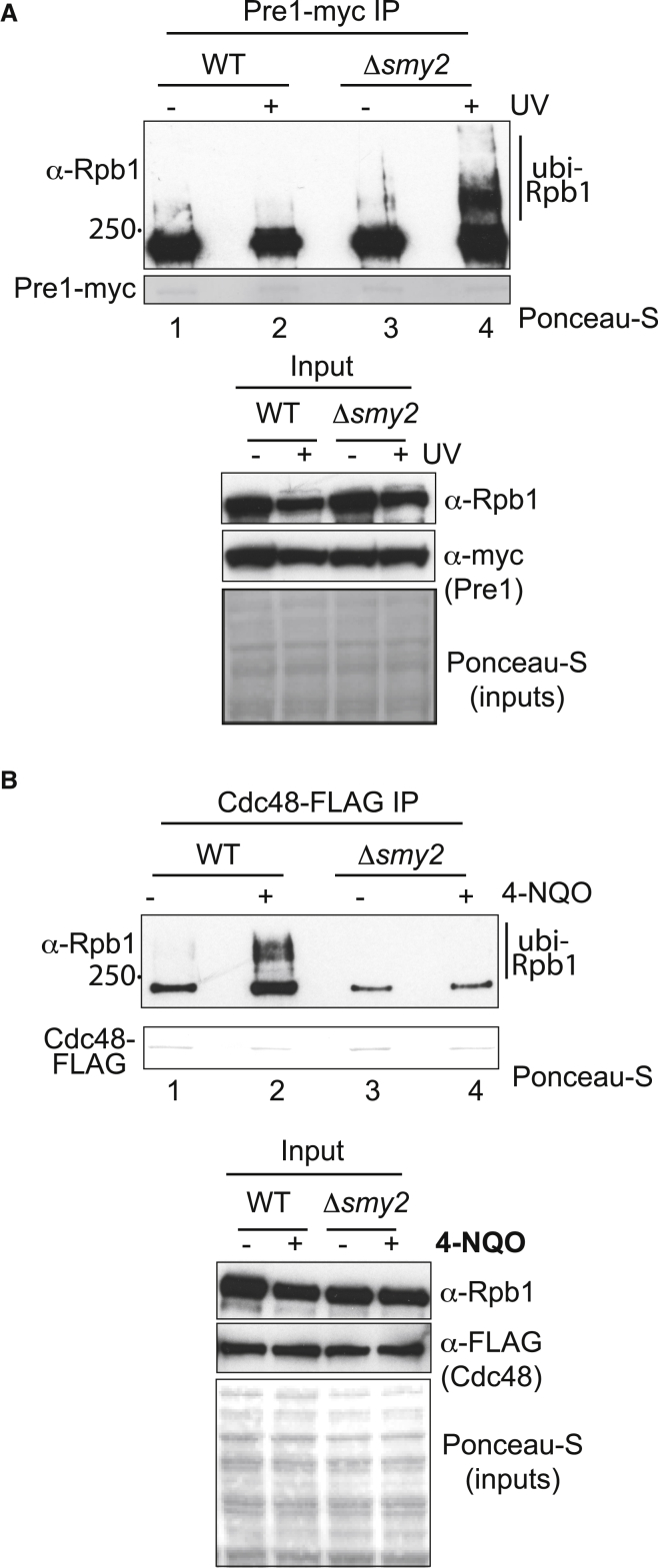
Smy2 connects Cdc48, ubiquitylated Rpb1, and the proteasome (A) Top, western blot showing effect of *SMY2* deletion on coIP of Rpb1 with the Myc-tagged proteasome subunit Pre1, before and after DNA damage. The Ponceau S stain shows Pre1 eluted from the beads. Bottom, inputs. Poly-ubiquitylated Rpb1 is indicated on the right. (B) Top, western blot showing effect of *SMY2* deletion on coIP of Rpb1 with FLAG-tagged Cdc48, before and after DNA damage. The Ponceau S stain shows Cdc48-FLAG from the beads. Bottom, inputs.

To evaluate whether Rpb1 similarly accumulates on proteasomes in UV-treated *smy2Δ* cells, we UV irradiated WT and *smy2Δ* cells expressing the proteasome subunit Pre1 with a C-terminal Myc_9_ tag, isolated proteasomes via anti-Myc beads, and immunoblotted for Rpb1 (Figure 4A). Previous experiments with cells lacking the Cdc48 co-factors Ubx4 or Ubx5 showed that these accumulate poly-ubiquitylated Rpb1 on the proteasome after DNA damage, seen as extremely slowly migrating Rpb1 species (Verma et al., 2011). There was a strong accumulation of such Rpb1 species on proteasomes isolated from UV-irradiated *smy2Δ* cells (Figure 4A, compare lanes 2 and 4).

The experiments above suggest that Smy2 functions with Cdc48 to extract Rpb1 from the RNAPII complex, possibly acting as a facilitator of the interaction between RNAPII and Cdc48. To test this possibility, we used congenic Cdc48-FLAG strains, one of which had a *SMY2* deletion (*smy2Δ*). We then exposed the cells to 4-NQO, isolated Cdc48 by FLAG IP, and immunoblotted for Rpb1. As expected from previous data showing that Cdc48 extrudes Rpb1 (Verma et al., 2011), poly-ubiquitylated Rpb1 is strongly coIPed with Cdc48-FLAG in WT cells after DNA damage (Figure 4B, compare lanes 1 and 2). Remarkably, however, in *smy2Δ* cells, little or no co-precipitation of Rpb1 with Cdc48 was observed (Figure 4B, lanes 3 and 4). These data are important, as they indicate that Smy2 is required for a strong interaction between poly-ubiquitylated Rpb1 and Cdc48 to help mediate the efficient extrusion of Rpb1; they thus provide further evidence for the idea that Smy2 helps facilitate Cdc48 function.

### A broad role for Smy2 in Cdc48-mediated processes

Our data thus far indicate that Smy2 functions in the transcription stress response as a regulator of Cdc48 recognition and possibly extrusion of Rpb1. Because the Ubx4 and -5 adaptor proteins work with Cdc48 in this and other processes (Verma et al., 2011), we tested if Smy2 has a similar broad function.

**Figure 5:**
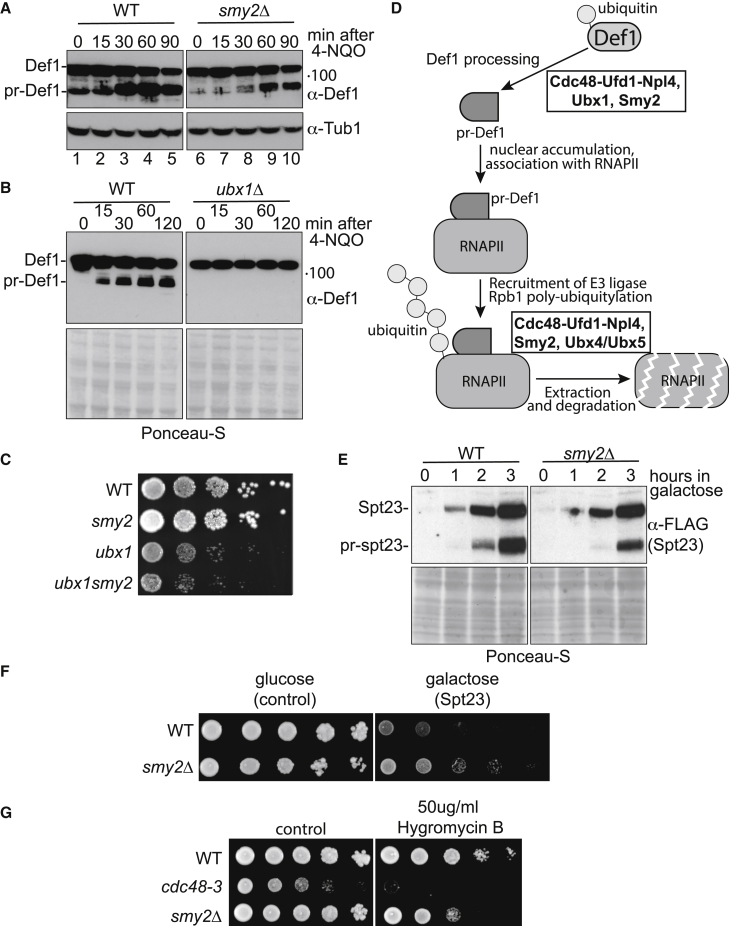
Smy2 and Ubx1 regulate processing of Def1 (A) Western blot of cell extracts showing the effect of *SMY2* deletion on processing of Def1, before and after DNA damage. Pr-Def1, processed Def1. Tubulin is loading control. (B) As in (A) but effect of *ubx1* deletion. Ponceau S stain provides loading control. (C) Dilution series of yeast cells lacking *SMY2*, *UBX1*, or both, grown for 2–4 days. (D) Model for the effect of Cdc48, Smy2, and different Ubx proteins in the transcription stress response. Only the Rpb1 subunit of the RNAPII complex is actually degraded by the proteasome. (E) Western blot showing effect of *SMY2* deletion on processing of FLAG_3_-Spt23, expressed from the *GAL* promoter. Pr-spt23, processed Spt23. (F) Dilution series of WT and *smy2*Δ cells containing a plasmid expressing *GAL*-inducible FLAG_3_-Spt23, plated onto minimal media containing glucose or galactose. (G) Dilution series of yeast cells showing the effect on growth of *SMY1* deletion and *cdc48-3* mutation in the presence of hygromycin B. See also Figure S2.

Cdc48 is also required for the proteasome- and ubiquitin-dependent processing of Spt23 and Mga2 (Hoppe et al., 2000; Rape et al., 2001; Shcherbik and Haines, 2007; Kolawa et al., 2013). We hypothesized that Smy2 might play an auxiliary role in such Cdc48-dependent processing as well. First, we investigated if Smy2 is required for the proteasomal processing of Def1, which is triggered by mono-ubiquitylation by Rsp5 (Wilson et al., 2012, 2013b). The active, processed form of Def1 (pr-Def1) accumulates in the nucleus to stimulate the interaction between mono-ubiquitylated Rpb1 and the Elongin-cullin E3 ligase complex for Rpb1 poly-ubiquitylation (Wilson et al., 2013b). WT and *smy2Δ* cells were subjected to 4-NQO treatment, and the cell lysates were immunoblotted for Def1. As can be seen in Figure 5A, the faster-migrating Def1 form, pr-Def1, was greatly induced, as previously reported (Wilson et al., 2013b). However, in *smy2Δ* cells, pr-Def1 appeared at later time points and in much reduced amounts relative to full-length Def1 (Figure 5A, compare lanes 8–10 with 3–5). This suggests that Smy2 also affects Def1 processing.

Cdc48 with its Ufd1-Npl4 adapter requires the assistance of a UBX protein for efficient proteasomal processing of both Spt23 and Mga2 (Kolawa et al., 2013). We therefore also tested if proteasomal processing of Def1 similarly requires a Ubx protein. Interestingly, UV-induced Def1 processing was only fully deficient in one *ubx*-deficient strain, namely *ubx1Δ* (Figures 5B and S2). Tellingly, we observed a synthetic growth defect in *ubx1Δ smy2Δ* mutant cells compared with the single *ubx1Δ* and *smy2Δ* mutants (Figure 5C). Previous data by Deshaies and co-workers showed that among the *ubx* mutants, *ubx1Δ*, *ubx4Δ*, and *ubx5Δ* all affect UV-induced Rpb1 degradation. However, only *ubx4Δ* and *ubx5Δ* cells accumulate poly-ubiquitylated Rpb1 (Verma et al., 2011). The puzzling lack of accumulation of poly-ubiquitylated Rpb1 in *ubx1* mutants may now be explained (Figure 5D): we propose that *ubx1Δ* cells fail to accumulate poly-ubiquitylated Rpb1 on the proteasome because Ubx1 is required upstream—for Def1 processing—which is in turn required for ubiquitylation of the polymerase to occur (Woudstra et al., 2002; Wilson et al., 2013b). More generally, our data support a model whereby Smy2 somehow works alongside the Ubx adaptor proteins (and the Ufd1/Npl4 co-factors) rather than in a mutually exclusive fashion. In the transcription stress response leading to Rpb1 ubiquitylation and degradation (Wilson et al., 2013a), Cdc48, Smy2, and Ubx proteins thus appear to function together in two different steps. First, Smy2 works with Ubx1 and Cdc48 for efficient proteasome-mediated Def1 processing and thereby supports ubiquitylation of Rpb1, and secondly, Smy2 also works with Cdc48 and Ubx4/5 for extrusion of ubiquitylated Rpb1 from the damage-stalled RNAPII complex in chromatin (summarized in Figure 5D).

The Smy2-regulated processes studied above are all associated with transcription stress and the last resort pathway. We now investigated if Smy2 might also work with Cdc48 in a separate cellular process, namely in the proteasomal processing of the transcriptional activator Spt23, which is extruded from the endoplasmic reticulum (ER) in a Cdc48-dependent process (Auld and Silver, 2006). Briefly, during unsaturated fatty acid synthesis, the ER transmembrane-associated form of Spt23 (p120) is ubiquitylated and then processed/clipped by the proteasome. In a Cdc48-dependent reaction, this releases a processed Spt23 p90 form from its unprocessed p120 partner in the ER membrane, allowing it to translocate to the nucleus, where it activates transcription of the *OLE1* gene (Rape et al., 2001). *OLE1* in turn encodes stearoyl-Δ9 desaturase, which governs the conversion of saturated fatty acids to unsaturated fatty acids. In previous work, the laboratories of Jentch, Haines, Deshaies, and others established a role for not only Cdc48 but also its regulatory factors Ufd1, Npl4, and Ubx2 in the processing and release of Spt23 from the ER (see, for example, Hoppe et al., 2000; Rape et al., 2001; Kolawa et al., 2013; Verma et al., 2013). We tested whether Smy2 also functions in this pathway by comparing the processing of inducible Spt23 (FLAG_3_-Spt23-HA; Kolawa et al., 2013) in WT and *smy2Δ* cells. Galactose was added at time 0 to induce expression of Spt23, and samples were collected hourly for 3 h to investigate the appearance of the full-length and processed forms by immunoblotting (Figure 5E). GAL induction of *SPT23* (the p120 form) was not noticeably affected by the lack of *SMY2*. However, a defect in Spt23 p90 production was observed in *smy2Δ* cells (Figure 5E, compare the 2-h time points, for example), indicating that Smy2 affects the Cdc48-regulated Spt23 pathway as well.

To investigate the physiological importance of this defect, we examined the effect of GAL-induced *SPT23* over-expression on the growth of *smy2Δ* cells. While unsaturated fatty acids are required for survival, excess production of oleic acid after hyperactivation of *OLE1* in response to Spt23 over-expression is toxic in WT cells, but *cdc48-3* and *ubx2Δ* cells tolerate such over-expression because they fail to support efficient Spt23 processing and p90 accumulation (Kolawa et al., 2013). Significantly, *smy2Δ* cells were much less affected than WT by over-expression of Spt23 as well (Figure 5F). Taken together, these data are consistent with Smy2 also functioning with Cdc48 and Ubx2 in proteasomal protein processing.

In another separate process, Cdc48 also promotes degradation of aberrant nascent polypeptides bound to the ribosome (Brandman and Hegde, 2016), and *cdc48-3* cells are sensitive to the translation inhibitor hygromycin B (Verma et al., 2013). Growth analysis demonstrated that although WT cells showed little, if any, growth defects in the presence of hygromycin B, not only *cdc48-3* but, more importantly, also *smy2Δ* cells were sensitive to the drug (Figure 5G).

Taken together, the data presented above on transcription stress and other cellular processes support the idea that Smy2 is a general regulator of the multi-functional Cdc48 protein in yeast.

### The human homologs of Smy2, GIGYF1 and -2, work with p97/VCP, the human homolog of Cdc48

**Figure 6:**
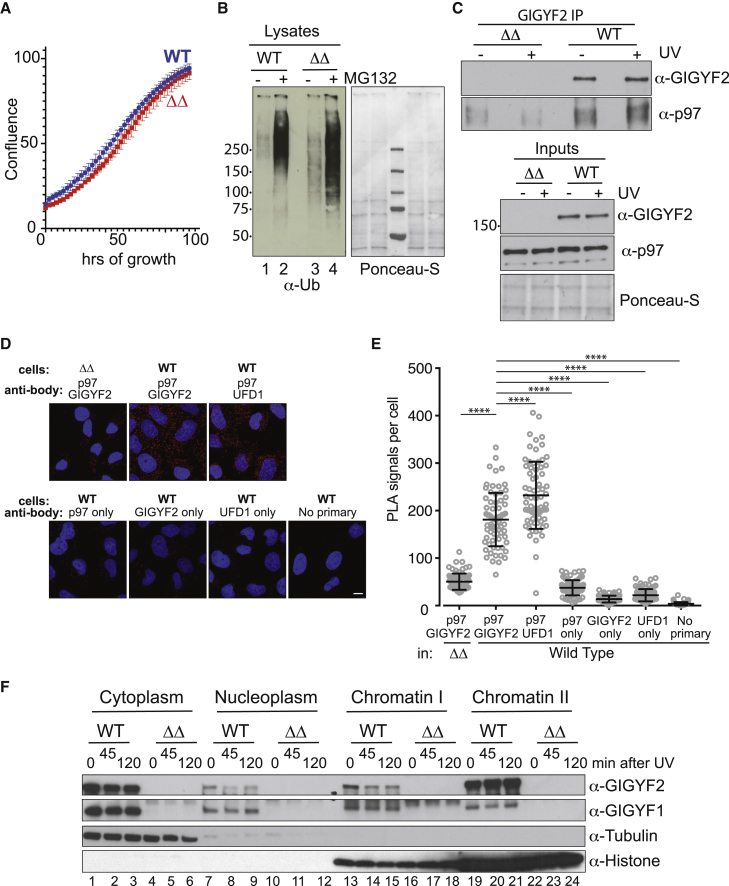
GIGYF2 protein interacts with p97/VCP, and *GIGYF* deletion affects the UPS in human cells (A) Growth of cells lacking both *GIGYF1* and *2* (*ΔΔ*), measured in Incucyte. Representative of 3 biological replicates; data are average of 3 independent wells. (B) Western blot showing the effect of *GIGYF1/2* double deletion (ΔΔ) on the level of poly-ubiquitylated proteins in the absence and presence of proteasome inhibitor (MG132), before or after UV irradiation. (C) Top, western blot of coIP of p97/VCP with GIGYF2 from WT or *GIGYF1/2* double-knockout cells (ΔΔ). Bottom, inputs. (D) Representative images from proximity ligation assays (PLAs), using the cells and antibodies indicated. White scale bar: 10 μm. (E) Graph showing PLA signals per cell from (D) in WT or *ΔΔ* cells with antibody combinations shown below. Mean and SD shown in black. ^∗∗∗∗^p < 0.0001 following one-way ANOVA and Dunnett’s multiple comparisons. 69 cells analyzed in each condition. Representative of 2 biological replicates. (F) Western blot of GIGYF1 and 2 after subcellular fractionation. Tubulin and histone mark the cytoplasm and chromatin, respectively.

Smy2 has two homologs in humans: GIGYF1 and GIGYF2. To determine if these proteins function in a manner similar to Smy2, we used CRISPR technology to generate a MRC5VA cell line in which both the *GIGYF1* and *GIGYF2* genes were knocked out (Figure S3A). These double-knockout cells (*ΔΔ*) grew normally (Figure 6A) and were visually indistinguishable from parental cells (see below). To first study the effect of *GIGYF1/2* KO on ubiquitin homeostasis, ubiquitylated proteins were analyzed by western blot analysis. This revealed an accumulation of ubiquitin conjugates in ΔΔ cells, which was exacerbated by incubation with the proteasome inhibitor MG132 (Figure 6B, compare lanes 1 and 3 and lanes 2 and 4).

To investigate if GIGYF2 interacts with p97/VCP, we performed GIGYF2 IP experiments from WT and *ΔΔ* cell extracts (Figure 6C). p97 was co-enriched with GIGYF2 from WT lysates but not from the *ΔΔ* lysates, and the interaction appeared to be independent of UV irradiation, as in yeast. As a further test of the interaction between GIGYF2 and p97, we also performed proximity ligation assays (Fredriksson et al., 2002). In this approach, protein-protein interactions inside cells are observed as quantifiable punctate staining. WT cells concurrently incubated with both GIGYF2 and p97/VCP antibodies showed an interaction, which was comparable to that between p97 and its well-known regulator UFD1 (Figure 6D, quantification in Figure 6E). In contrast, *ΔΔ* cells tested in the same manner failed to show interaction, and other controls displayed no significant signal either. Together, these data indicate that GIGYF2 and p97 interact in human cells.

To determine the subcellular localization of GIGYF1 and GIGYF2, we performed fractionation of WT and ΔΔ knockout cell extracts. Cells were fractionated before or after exposure to UV, with recovery for the indicated times. A significant amount of both GIGYF1 and GIGYF2 is present in the cytoplasm (Figure 6F, lanes 1–3). This was expected, as GIGYF2 has a role in the control of protein translation (Morita et al., 2012; Tollenaere et al., 2019; Hickey et al., 2020; Juszkiewicz et al., 2020). Importantly, however, while only a relatively minor amount of the GIGYF proteins was observed in the nucleoplasm (lanes 7–9), both proteins, but especially GIGYF2, were tightly associated with chromatin (Figure 6F, chromatin II, lanes 19–21).

**Figure 7:**
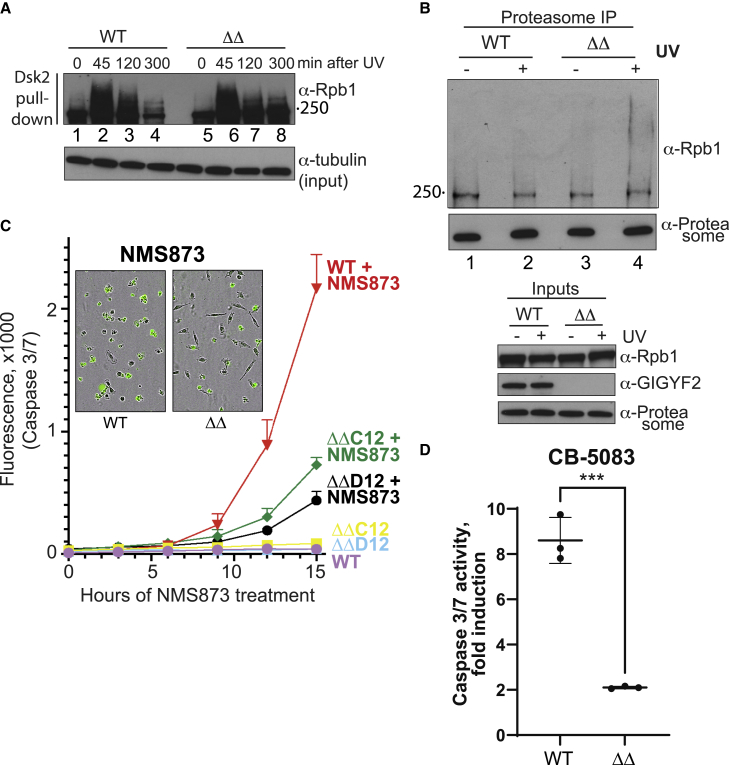
*GIGYF* deletion affects the transcription stress response and has global effects on p97/VCP function in human cells (A) Western blot of ubiquitylated RPB1 from WT and ΔΔ cells before or after treatment with UV and recovery for the indicated times, followed by GST-Dsk2 pull down of ubiquitylated proteins. Tubulin is indicator for inputs. (B) Western of RPB1 coIPed with proteasomes from WT and *ΔΔ* cells before and after DNA damage. (C) The apoptosis-inducing effect of p97/VCP inhibitor NMS873 and its dependence on the GIGYF proteins. Two ΔΔ clones tested (C12 and D12). Representative of 3 biological replicates; data from 4 independent wells in each condition. Data are expressed as the mean ± standard deviation (SD) of the mean, and statistical analysis was carried out using PRISM (GraphPad) software. (D) Same as (C) but testing the p97/VCP inhibitor CB-5083 15 h after adding inhibitor. Only clone C12 tested. Data from 3 biological replicates; data are average of 3 independent wells in each condition in each experiment. Results were analyzed using GraphPad Prism 9 software, and statistical analysis by the unpaired t test showed a p value of 0.0004. See also Figure S3.

Given the results obtained in yeast, we hypothesized that GIGYF1/2 knockout cells would have a defect in extracting ubiquitylated RPB1. To investigate this possibility, we collected lysates from WT and ΔΔ cells at different time points after UV irradiation. Ubiquitylated proteins were then isolated by incubation with immobilized GST-Dsk2 (Tufegdzic Vidakovic et al., 2019). The ΔΔ cells indeed showed evidence of somewhat slower clearance of ubiquitylated RNAPII (Figure 7A, compare lanes 4 and 8). To test the hypothesis that *GIGYF* deletion affects the proteasomal processing of ubiquitylated proteins such as RNAPII, we investigated if there was an increase in ubiquitylated RPB1 associated with the proteasome in *ΔΔ* cells exposed to UV. The proteasome was IPed from solubilized chromatin, and co-precipitated RPB1 was analyzed by western blotting. Compared with WT, ΔΔ cells showed clear accumulation of ubiquitylated RPB1 when exposed to UV (Figure 7B, compare lanes 2 and 4). These data are again reminiscent of those obtained in yeast (Figure 4) and further support a model in which Smy2 and GIGYF1/2 act as a bridge between Cdc48/p97 and polyubiquitylated proteins, including RNAPII.

Our data in yeast suggested a broad involvement of Smy2 with Cdc48 beyond that in transcription stress, so to investigate the connection between p97/VCP and GIGYF proteins more generally, we finally tested the effect of chemical inhibitors. NMS-873 is an allosteric p97/VCP inhibitor, which is known to activate the unfolded protein response, interfere with autophagy, and to induce cancer cell death through apoptosis (Magnaghi et al., 2013). Presumably, the broad effect on cell function and viability reflects NMS-873’s interference with various cell pathways that rely on correctly regulated p97/VCP function. WT and two different GIGYF ΔΔ cell lines (C12 and D12) were treated with NMS-873, and the level of apoptosis investigated. Remarkably, while WT cells, as expected, showed dramatic increases in apoptosis upon p97/VCP inhibition, the effect was markedly reduced in the ΔΔ cells (Figures 7C and S3B). A distinct p97/VCP drug, CB-5083 (Zhou et al., 2015), also affected WT cells much more than cells lacking the GIGYF proteins (Figure 7D). Together, these results indicate that the GIGYF proteins are required for the function of a well-known p97/VCP drug and attest to the general importance of the GIGYF proteins for p97/VCP biology.

## Discussion

Many of the proteins required for the cellular response to transcription stress also play other roles in the UPS. Indeed, the last resort pathway, which facilitates ubiquitylation and degradation of the largest RNAPII subunit, Rpb1, involves multi-functional ubiquitin ligases and ubiquitin proteases, as well as Cdc48/p97/VCP and the proteasome (Wilson et al., 2013a; Gregersen and Svejstrup, 2018). In our search for additional constituents, we isolated the yeast *SMY2* gene as a multi-copy suppressor of deletion of *DEF1*, a key member of the last resort pathway. In this report, we provide evidence from genetic analysis and molecular cell biology experiments that not only Smy2, but also its human homologs GIGYF1 and -2, are involved in the transcription stress response as regulators of Cdc48/VCP function. Furthermore, our results suggest that these proteins may play a more general role in regulating Cdc48/VCP function also in other cellular processes.

### Involvement of Smy2/GIGYF proteins in the transcription stress response and, more generally, as a regulator of Cdc48/VCP function

In response to insults such as DNA damage, the yeast Def1 protein is processed by the proteasome and accumulates in the nucleus, which enables recruitment of the Elc1-Ela1-Cul3 ligase complex to damage-stalled RNAPII (Wilson et al., 2013b). Ubiquitylation is a two-step process, with the Rpb1 poly-ubiquitylation by this complex requiring prior (mono-)ubiquitylation by ubiquitin ligase Rsp5 (Huibregtse et al., 1997; Beaudenon et al., 1999; Somesh et al., 2007; Harreman et al., 2009). Poly-ubiquitylated RNAPII is then targeted by the proteasome, but efficient proteolysis requires Cdc48 (Verma et al., 2011). Indeed, ubiquitylated Rpb1 accumulates on the proteasome in the absence of Cdc48, regulators of Cdc48 function such as Ubx4 or -5, or the chromatin remodeler INO80 (Verma et al., 2011; Lafon et al., 2015). The process is highly conserved, with homologs of the yeast proteins involved in the process in human cells (Anindya et al., 2007; Yasukawa et al., 2008; Harreman et al., 2009; Wilson et al., 2013a; Weems et al., 2017).

Smy2, and its human homologs GIGYF1/2, can now be added to this disparate set of transcription stress factors. In cells lacking Smy2, ubiquitylated Rpb1 accumulates on the proteasome. Importantly, the ability of Cdc48 to associate with ubiquitylated Rpb1 to enable its extraction (Verma et al., 2011) is dramatically diminished in *smy2Δ* cells, indicating that Smy2 helps direct Cdc48-mediated Rpb1 extraction. Intriguingly, Smy2 appears to generally regulate Cdc48 function: Smy2 and Cdc48 interact, and over-expression of *SMY2* suppresses the growth defect of different *cdc48* temperature-sensitive strains. Likewise, while *smy2Δ* cells show no growth defect, *cdc48-3 smy2Δ* double mutants grow slower than *cdc48-3* at a semi-restrictive temperature. Smy2 also appears to affect Cdc48-facilitated reactions more generally, with ubiquitylated proteins accumulating and Cdc48-dependent proteasome-mediated processing of Def1 and Spt23 also incomplete in *smy2*Δ cells.

Importantly, the results in yeast are mirrored by those obtained in human cells: here, double knockout of the genes encoding the two closely related Smy2 homologs GIGYF1 and 2 also results in an accumulation of ubiquitylated RPB1 on proteasomes and in a general accumulation of ubiquitylated proteins. Such an accumulation has previously been reported for cells lacking the *D. melanogaster* GIGYF1/2 homolog, Gyf/GRB10, though without the underlying mechanism having been investigated (Kim et al., 2015). GIGYF2 protein interacts with p97/VCP, suggesting that, like in yeast, the effect of GIGYF deficiency may be explained by an effect on p97/VCP function. This notion is strongly supported by experiments with chemical VCP inhibitors, the effects of which are dramatically abated in cells lacking the GIGYF proteins.

### The putative role of Smy2/GIGYF proteins as regulators of Cdc48/VCP function

As outlined above, the data to support the idea that Smy2 (and thus GIGYF proteins) function in the transcription stress response in the context of Cdc48 function are compelling. Most importantly, deletion of *SMY2* has effects that are strikingly similar to those of *cdc48 and ubx4/5* deficiency in this response (Verma et al., 2011), and Cdc48 interaction with ubiquitylated Rpb1 is dramatically decreased in *smy2Δ* cells after DNA damage.

Smy2 and GIGYF2 play roles in several distinct cellular processes (Higashio et al., 2008; Sezen et al., 2009; Morita et al., 2012; Peter et al., 2017; Hickey et al., 2020; Juszkiewicz et al., 2020; Sinha et al., 2020; Nordgaard et al., 2021). It is possible that Smy2 and GIGYF2 also have Cdc48/VCP-independent roles or that Cdc48/VCP plays a hitherto unrecognized role in processes that were shown to be Smy2/GIGYF regulated. As a putative example of this, *SMY2* was identified in a genetic screen for factors involved in coat protein complex II (COPII) vesicle formation (Higashio et al., 2008). COPII is essential for transport vesicle formation from the ER and is composed of two heterodimeric subcomplexes, Sec23p/Sec24p and Sec13p/Sec31p, and the small guanosine triphosphatase Sar1p (Jensen and Schekman, 2011). *SMY2* is a multi-copy suppressor of the temperature-sensitive *sec24-20* mutant and exhibits genetic interactions with several other genes involved in ER-to-Golgi transport. Smy2 interacts with the Sec23p/Sec24p subcomplex (Higashio et al., 2008). Interestingly, other data show that Sec23 is ubiquitylated by Rsp5 and de-ubiquitylated by Usp3/Bre5, with the ubiquitylated form of Sec23 unable to interact with Sec24. The ubiquitylated form of Sec23 is degraded by the proteasome (Cohen et al., 2003; Ossareh-Nazari et al., 2010). While the precise role played by Smy2 in this process remains unclear, we note that Cdc48 is required for the proteasomal degradation of ubiquitylated Sec23 (Ossareh-Nazari et al., 2010), providing a connection between Sec23, Smy2, and Cdc48 in the regulation of COPII vesicle formation.

Likewise, GIGYF2 inhibits mRNA translation through an interaction with the alternative cap-binding protein EIF4E2, which outcompetes the normal cap-binding protein eIF4E to prevent new initiation (Cho et al., 2005; Morita et al., 2012; Chapat et al., 2017; Peter et al., 2017; Tollenaere et al., 2019). Budding yeast does not have a recognizable EIF4E2 homolog, but Smy2 interacts with Eap1, an inhibitor of eIF4E function (Sezen et al., 2009), suggesting that the process is conserved. Interestingly, recent results indicate that inhibition of translational initiation in human cells can be triggered *in cis* by problems during translational elongation of the same mRNA transcript, sensed by the ribosome quality control complex (RQC) (Hickey et al., 2020; Juszkiewicz et al., 2020; Sinha et al., 2020). Intriguingly, yeast RQC comprises the Ltn1 E3 ubiquitin ligase, Tae2, and Rqc1, as well as Cdc48 and its co-factors Npl4 and Ufd1 (Brandman et al., 2012). In human cells, VCP is part of the analogous RQC pathway (see, for example, Shao and Hegde, 2014). Whether VCP and Cdc48 play a direct role in the GIGYF-EIF4E2 (Smy2-Eap1) pathways for inhibition of translation inhibition remains to be determined, but VCP/Cdc48 is certainly connected with the process.

While we suggest that Smy2/GIGYF proteins may function as general regulators of Cdc48/VCP function, future work will be required to investigate the precise molecular mechanism. Smy2 appears to function alongside the Ubx proteins, not in place of them. Our experiments indicate that Smy2 and Cdc48 interact. However, it remains to be investigated whether Smy2/GIGYF interacts with Cdc48/VCP/p97 only via the GYF motif and if and how it interacts with target proteins to regulate Cdc48/VCP/p97 function. We note that characterization of the GIGYF2 protein showed that it uses distinct domains to interact with 4EHP and DDX6, for example (Morita et al., 2012; Peter et al., 2017, 2019). We also note that the Smy2 IP experiments indicate that it (also) interacts with un-ubiquitylated RNAPII, suggesting that it does not provide the specificity for Cdc48 to recognize the ubiquitylated Rpb1 form. Instead, we suggest that Cdc48 or one of its Ubx adaptor proteins provides this function, while Smy2 enables generally stronger RNAPII association.

### GIGYF proteins affect VCP inhibitor function

The dramatic effect of *GIGYF1/2* deletion on the functionality of two distinct and specific VCP inhibitors, NMS-873 (Magnaghi et al., 2013) and CB-5083 (Zhou et al., 2015), provides strong evidence for a close functional connection between GIGYF proteins and VCP/p97. At first glance, it seems surprising and counter-intuitive that these VCP inhibitors work less well in cells lacking the GIGYF proteins. We can thus only speculate on the explanation. However, it is important to know that NMS-873 and CB-5083 are VCP/p97 ATPase inhibitors; they affect VCP’s ability to function only after it is engaged with a target protein. Indeed, ATP hydrolysis is required for the release/turnover of Cdc48 from ubiquitylated target proteins substrates *in vitro* (Bodnar and Rapoport, 2017), and both VCP and ubiquitylated proteins accumulate on mammalian chromatin in the presence of VCP inhibitor CB-5083 (Rycenga et al., 2019). It is thus likely that these inhibitors are deleterious first and foremost because they catch an intermediate in the catalytic cycle of VCP, in effect immobilizing VCP on target proteins, with detrimental effects that trigger apoptosis. Removal of factors that enable efficient association of VCP with its targets in the first place would thus be expected to reduce inhibitor-induced cell death. In this model, the deletion of *GIGYF* genes would have the effect of generally decreasing VCP engagement with its cellular targets, thus reducing the detrimental immobilizing effects of inhibiting its ATPase activity and thus reducing apoptosis. Alternatively, the GIGYF proteins might affect stress induction and cell death signaling downstream of Cdc48 function, but this, in our opinion, seems less likely considering the results from yeast genetics.

### Limitations of the study

While the results from genetic and functional approaches presented here all support a role for Smy2/GIGYF proteins in the regulation of Cdc48/VCP function, it is not clear whether Smy2/GIGYF work as direct adaptors/regulators or merely affect the same disparate pathways and thus work indirectly. Additional biochemical and molecular cell biology approaches will thus be required to delineate the precise functional relationship between Smy2/GIGYF and Cdc48/VCP.

## STAR★Methods

### Key resources table

**Table:** 

REAGENT or RESOURCE	SOURCE	IDENTIFIER
**Antibodies**		

Mouse monoclonal anti-Rpb1	Abcam	Cat#ab5408; RRID: AB_304868
Mouse monoclonal anti-ubiquitin (P4D1)	ENZO Life Sciences	Cat#BML-PW0930; RRID: AB_10998070
Mouse monoclonal anti-ubiquitin (P4G7)	ENZO Life Sciences	Cat#ENZ-ABS142; RRID: AB_2331077
Mouse monoclonal anti-HA	Santa Cruz	Cat#1424; RRID: AB_301017
Mouse monoclonal anti-GST	Abcam	Cat#sc-516102; RRID: AB_2687626
Rabbit polyclonal anti-FLAG	Sigma	Cat#F7425; RRID: AB_439687
Mouse monoclonal anti-MYC (9E10)	Crick Laboratories	Evan et al. (1985)
Rabbit polyclonal anti-Def1 (388–738)	Crick Laboratories	Wilson et al., 2013a, 2013b
Mouse monoclonal anti-tubulin	Sigma	Cat#T5168, RRID: AB_477579
Rabbit monoclonal anti-proteasome 20S C2/HC2	Abcam	Cat#ab109530; RRID: AB_10860339
Rabbit polyclonal anti-histone H3	Abcam	Cat#ab18521; RRID:AB_732917
Rabbit polyclonal anti-GIGYF2	Bethyl	Cat#A303-731A; RRID: AB_11204927
Rabbit polyclonal anti-GIGYF2	Proteintech	Cat#24790-1-AP; RRID: AB_2879727
Rabbit polyclonal anti-GIGYF1	Bethyl	Cat#A304-132A; RRID: AB_2621381
Rabbit polyclonal anti-UFD1L	Proteintech	Cat#10615-1; RRID: AB_2213944
Rabbit monoclonal anti-VCP	Abcam	Cat#ab109240; RRID: AB_10862588
Mouse monoclonal anti-VCP	Abcam	Cat#ab11433; RRID: AB_298039
Anti-rabbit secondary (HRP)	Jackson ImmunoResearch	Cat# 711-035-152; RRID: AB_10015282
Anti-mouse secondary (HRP)	Jackson ImmunoResearch	Cat# 715-035-151 RRID: AB_2340771
Anti-mouse secondary (HRP)	Santa Cruz	Cat# sc-516102; RRID: AB_2687626

**Bacterial and virus strains**		

NEB 5-alpha Competent E. coli (High Efficiency)	New England Biolabs	Cat#C2987H
BL21-CodonPlus (DE3)-RIL Competent Cells	Agilent technologies LDA UK LTD	Cat#230245

**Chemicals, peptides, and recombinant proteins**		

lipofectamine 3000	Life Technologies	Cat#L3000015
NMS873	Tebu-Bio Ltd.	Cat#282T1853
CB-5083	Cayman Chemicals	Cat#CAY19311
Dimethyl Sulfoxide (DMSO)	Sigma-Aldrich	Cat#D2650
N-Ethylmaleimide (NEM)	Sigma-Aldrich	Cat#04260
Ampicillin	Cambridge Bioscience limited	Cat#2484
MG-132	Sigma-Aldrich	Cat#M7449
6-azauracil	Sigma-Aldrich	Cat#A1757
5-fluoroorotic acid	Melford Laboratories	Cat#F5001-5G
Dsk2 beads	Home-made; see Tufegdzic Vidakovic et al., 2019	N/A
Hygromycin B	Enzo Life Sciences	ALX-380-306-G001

**Critical commercial assays**		

Duolink Kit *in situ* Red starter kit mouse/rabbit	Sigma-Aldrich	Cat#DUO92101
Caspase-Glo 3/7 assay reagent	Promega Corporation	Cat#G8090
NucView 488 Caspase-3 Assay Kit	Biotium	Cat#BT30029-T
XT-sample buffer	BioRad Laboratories	Cat#1610791
4-15% TGX gels	BioRad Laboratories	Cat#5671084
NuPAGE 10% Bis-Tris protein gels	Life Technologies LTD	Cat#NP03031BOX
Criterion XT Tris-Acetate Gel 3–8%	BioRad laboratories	Cat#3450130
Criterion XT Bis-Tris 4-12%	BioRad Laboratories	Cat#3450125
Nitrocellulose membrane 0.45 uM	GE Healthcare Life Sciences	Cat#10600002
Nitrocellulose membrane 0.2 uM	GE Healthcare Life Sciences	Cat#10600019
Hyperfilm ECL	VWR international	Cat#29-9068-37
SuperSignal West Pico PLUS ECL reagent	Thermo Scientific	Cat#34580
DMEM media	Thermo Scientific	Cat#41966029
XT Tricine running Buffer	BIO-RAD Laboratories	Cat#1610790
Basemuncher	EXPEDEON LTD	Cat#BM0100
Glutathione Sepharose 4B	Sigma-Aldrich	Cat#GE17-0756-01
ANTI-FLAG M2 Affinity Gel	Sigma-Aldrich	Cat#A2220
Protein G agarose beads	Sigma-Aldrich	Cat#11719416001
Complete EDTA-free protease inhibitor cocktail	Sigma-Aldrich	Cat#05056489001
PhosSTOP	Sigma-Aldrich	Cat#04906837001
SilverQuest Silver Staining Kit	Thermo Fisher	Cat#LC6070
Ponceau S	Sigma-Aldrich	Cat# P7170
Bio-Rad protein assay reagent	Bio-Rad	Cat#5000006
Precision PLUS pre-stained markers	BioRad Laboratories	Cat#1610393

**Experimental models: Cell lines**		

Human lung fibroblast cell line MRC5VA	Francis Crick Institute cell depository	N/A
Human lung fibroblast cell line MRC5VA GIGYF1 and 2 KO	Francis Crick Institute cell depository	N/A

**Experimental models: Organisms/Strains**		

*S*.*cerevisiae* (strain W303 MATα ura3, leu2-3, 112, his3-11,15 trp1-1, ade2-1, can1-100)	Gift from R. Rothstein	N/A
*S*.*cerevisiae* (strain W303 MATa ura3, leu2-3, 112, his3-11,15 trp1-1, ade2-1, can1-100)	Gift from R. Rothstein	N/A
*S*.*cerevisiae* W303 MATa def1::TRP	Woudstra et al., 2002	N/A
*S*.*cerevisiae* W303 MATa rad16::LEU	Woudstra et al., 2002	N/A
*S*.*cerevisiae* W303 MATa def1::TRP rad16::LEU	Woudstra et al., 2002	N/A
*S*.*cerevisiae* W303 MATa rpo21::ADE2 (pJS121; RPO21 k330r TRP CEN)	Somesh et al. (2007)	N/A
*S*.*cerevisiae* W303 MATa rpo21::ADE2 (pJS121; RPO21 k330r TRP CEN) def1::URA	This manuscript	N/A
*S*.*cerevisiae* W303 MATa myc_9_-TEV_2_-His_6_-Smy2	This manuscript	N/A
*S*.*cerevisiae* W303 MATa galactose-FLAG-Smy2	This manuscript	N/A
*S*.*cerevisiae* W303 MATa myc_9_-TEV_2_-His_6_-Smy2 Cdc48-HA	This manuscript	N/A
*S*.*cerevisiae* W303 cdc48-3	Cheng and Chen (2010)	N/A
*S*.*cerevisiae* W303 cdc48-3 Smy2::TRP	This manuscript	N/A
*S*.*cerevisiae* W303 Pre1-myc_9_	This manuscript	N/A
*S*.*cerevisiae* W303 Pre1-myc_9_ Smy2::HIS	This manuscript	N/A
*S*.*cerevisiae* W303 Smy2::HIS	This manuscript	N/A
*S*.*cerevisiae* W303 Smy2::LEU	This manuscript	N/A
*S*.*cerevisiae* W303 Smy2::TRP	This manuscript	N/A
*S*.*cerevisiae* W303 CDC48-FLAG_3_	This manuscript	N/A
*S*.*cerevisiae* W303 CDC48-FLAG_3_ Smy2::LEU	This manuscript	N/A
*S*.*cerevisiae Y7731* MATa cdc48-9::KanR ura3Δ0 leu2Δ0 his3Δ met15Δ *cdc48-9*	Magtanong et al., 2011	N/A
*S*.*cerevisiae Y7829 cdc48-2* MATa cdc48-2::KanR ura3Δ0 leu2Δ0 his3Δ met15Δ	Magtanong et al., 2011	N/A
*S*.*cerevisiae* (strain BY4742, MATa, his3Δ1, leu2Δ0, lys2Δ0, ura3Δ0)	Open Biosystems	N/A
*S*.*cerevisiae* BY4742 ubx1Δ::KanMX	Open Biosystems	N/A
*S*.*cerevisiae* BY4742 ubx2Δ::KanMX	Open Biosystems	N/A
*S*.*cerevisiae* BY4742 ubx3Δ::KanMX	Open Biosystems	N/A
*S*.*cerevisiae* BY4742 ubx4Δ::KanMX	Open Biosystems	N/A
*S*.*cerevisiae* BY4742 ubx5Δ::KanMX	Open Biosystems	N/A
*S*.*cerevisiae* BY4742 ubx6Δ::KanMX	Open Biosystems	N/A
*S*.*cerevisiae* BY4742 ubx7Δ::KanMX	Open Biosystems	N/A
*S*.*cerevisiae* BY4742 smy2Δ::KanMX	Open Biosystems	N/A
*S*.*cerevisiae* BY4742 ubx1Δ::KanMX smy2::HIS	This manuscript	N/A

**Oligonucleotides**		

All oligonucleotides are listed in Table S2	N/A	N/A

**Recombinant DNA**		

pSpCas9(BB)-2A-GFP (PX458)	Addgene	Cat#48138
pDEF1 2 micron URA	This manuscript	N/A
pSMY2 2 micron URA	This manuscript	N/A
pSmy2 CEN LEU	This manuscript	N/A
pSMY2 2 micron LEU	This manuscript	N/A
pSMY2 Y234A 2 micron LEU	This manuscript	N/A
pGST 6p-1-GYF (205–261)	This manuscript	N/A
pGST 6p-1-GAF (205–261) Y234A	This manuscript	N/A
pGEX 6p-1	Sigma-Aldrich	Cat#GE28-9546-48
pCDC48 CEN URA	This manuscript	N/A
pGAL-FLAG_3_-Spt23-HA_3_ URA	This manuscript	N/A
pRS426 URA 2 micron	(Sikorski and Hieter, 1989)	N/A
pRS306 URA CEN	(Sikorski and Hieter, 1989)	N/A
pRS305 LEU CEN	(Sikorski and Hieter, 1989)	N/A
pRS425 LEU 2 micron	(Sikorski and Hieter, 1989)	N/A
pGEX3-Dsk2	Anindya et al. (2007)	N/A
pGST-MD	Wilson et al. (2012)	N/A

**Software and algorithms**		

TIDE	Brinkman et al., (2014)	http://tide.nki.nl/
Prism 9.2.0	GraphPad	https://www.graphpad.com
Perseus version 1.4.0.11	Tyanova et al. (2016)	N/A
FIJI	ImageJ	https://imagej.net/software/fiji/

**Other**		

Coulter Cell Counter	Beckman	N/A
Amersham Imager 600 (AI600)	GE life sciences	N/A
Incucyte	Essenbioscience	N/A
High-copy suppressor library YEP353	Zhang et al. (1998)	N/A

### Resource availability

#### Lead contact

Further information and requests for resources and reagents should be directed to and will be fulfilled by the Lead Contact, Jesper Svejstrup (jsvejstrup@sund.ku.dk).

#### Materials availability

Yeast strains and plasmids generated in this study will be distributed without restriction upon request. Mass spectrometry data generated in this work are available in Table S1.

### Experimental model and subject details

#### Yeast strains and culture conditions

Unless otherwise stated, all *Saccharomyces cerevisiae* strains used in this study are in the W303 or BY4742 background and were grown at 30°C in YPD media (1% yeast extract, 2% bactopeptone, and 2% glucose) and manipulated using standard techniques (Sherman, 1991). Genotypes of all yeast strains are provided in the key resources table.

#### Human cell lines and culture conditions

MRC5-VA cells are an SV40 transformed human lung fibrobast cell-line. Cells were grown in DMEM with high glucose plus pyruvate, 1% penicillin/streptomycin and 10% FBS. Cells were split 1:10 every 4 days. Cell lines generated in the study:

MRC5-VA cells.

MRC5-VA *GIGYF1* KO.

MRC5-VA *GIGYF2* KO.

MRC5-VA *GIGYF1 GIGYF2* double KOs (C12 and D12)

All cell lines were authenticated, and confirmed to be mycoplasma-free by the Francis Crick Institute Cell Services.

#### Bacterial strains

NEB 5-alpha Competent E. coli (High Efficiency) (New England Biolabs) and BL21-CodonPlus (DE3)-RIL Competent Cells (Agilent technologies) were grown at 37°C, unless otherwise stated, in LB media (1% bactotryptone, 1% yeast extract and 0.5% sodium chloride) containing 75 μg/mL ampicillin.

### Method details

#### Plasmid construction

pDEF1 2-micron URA and pSMY2 2-micron URA were isolated from the high copy suppressor library (Zhang et al., 1998). and sequenced to confirm they were the only expressing ORF present in the each plasmid. pSMY2 2-micron LEU and pSmy2 CEN LEU were generated by amplifying the SMY2 ORF plus 1000 bp upstream and downstream with primers (Table S2) and cloning the product into pRS425 and pRS305 (Sikorski and Hieter, 1989), respectively. Site directed mutagenesis of pSMY2 2-micron LEU was used to create pSMY2 Y234A 2-micron LEU. A DNA fragment corresponding to the coding region of SMY2 amino acids 205–261 was subcloned into pGST 6p-1 (Sigma-Aldrich) to produce pGST 6p-1-GYF (205–261). Site directed mutagenesis of this plasmid was used to create pGST 6p-1-GAF (205–261) Y234A. pGAL-FLAG_3_-Spt23-HA URA was generated by amplifying the ORF of Spt23 with primers coding for FLAG_3_ (5′) and HA_3_ (3′) and cloning into pYES2 (Thermo Fisher). All plasmids were verified by sequencing.

#### Generation of stable cell lines

##### GIGYF1 and 2 knock-out cells

To generate *GIGYF 1* and *2* knock-out cells, MRC5VA cells were transfected with plasmid pX458 (Addgene) containing the indicated gRNA sequences (Benchling.com) using lipofectamine 3000 (Invitrogen, ThermoFisher Scientific). GFP-positive cells were selected by FACS and seeded into 96 well plates. Knock-outs were identified by Western Blotting using antibodies to GIGYF1 (A304-132) and GIGYF2 (A303-732), (Bethyl Laboratories Inc. Texas). Genomic DNA was then isolated on QiaAmp columns (Qiagen GmbH) and a region surrounding the edited site was sequenced and analyzed using TIDE software to confirm indel formation. All oligo sequences are described in Table S2.

##### Yeast high copy suppressor screen

High-copy suppressors of the benomyl-sensitive growth of *def1Δ* cells were identified by transforming cells with a YEp352-based high copy S. cerevisiae genomic library (Zhang et al., 1998). Transformants were grown on synthetic medium lacking uracil in the presence of benomyl at 25°C for 3-4 days to select for those plasmids able to complement the benomyl-sensitive growth phenotype. Suppression by each plasmid (plasmid linkage) was confirmed by rescuing each suppressor plasmid and then retransforming them into *def1Δ* cells to test for suppression of the slow growth on plates in the absence and presence of benomyl. Suppressors were identified by sequencing of the isolated suppressor plasmids genomic insert.

##### Yeast dilution series growth assays

Overnight yeast cultures were diluted to early logarithmic phase and grown for approximately 4 h. Cells were counted using a coulter counter and equal numbers serially diluted tenfold and spotted on the indicated agar plates. For 6-AU sensitivity assays, strains were made *URA +* by transformation with pRS316 (control) or the indicated plasmid on synthetic complete medium plates lacking uracil (SC−uracil), or SC−uracil plates containing 6-AU (50 μg/mL). Strains were tested for UV sensitivity on plates by irradiation with the indicated dose in a custom-made UV box as described below. Hygromycin sensitivity assays were carried out on YPD plates containing 50 μg/mL Hygromycin or YPD (control). Plates were incubated for 3–4 days at 30°C unless otherwise noted. After growth, the plates were photographed using a GelDoc XR (BioRad).

##### Mass spectrometric analysis

Peptides were generated by *in situ* tryptic digestion of gel bands. LC/MS/MS analysis of the peptides was performed by a Thermo LTQ-XL ion trap mass spectrometer, and resulting data searched against the SwissProt protein database by using the SEQUEST protein-searching algorithm, as previously described (Aygun et al., 2008).

##### UV-irradiation, MG132 and 4-NQO treatment

UV-irradiation was performed as previously described (Tufegdzic Vidakovic et al., 2019). Yeast cells were irradiated using a custom-made UV box with plates exposed to 6–25 J/m^2^ as indicated and liquid cultures with 300 J/m^2^ UV-light. For liquid yeast cultures, cells were collected, resuspended in one-fifth the volume of PBS and irradiated, recollected, and resuspended in the same media. MRC5VA-derived cells were irradiated with 20 J/m^2^. Media was removed, cells were irradiated using a custom-made UV conveyor belt and the same media replaced. UV doses were monitored using a UV meter (Progen Scientific) in all experiments. Where indicated, cells were treated with 5 μM MG132 for 1 h prior to UV irradiation. 4-NQO was added directly to the yeast medium and used at a final concentration of 8 μg/mL (10 mg/mL stock solution in ethanol) and incubated at 180 rpm for the noted times in a darkened incubator.

##### Purification of Myc_9_-TEV_2_-HIS_6_-Smy2

Yeast containing the Myc_9_-TEV_2_-HIS_6_-Smy2 protein at the endogenous locus were grown in YPD to a density of ∼6 × 10^7^ cells/mL. The cells were washed in ice-cold PBS, and the cell pellet was resuspended in an equal volume of extraction buffer (150 mM Tris-acetate, 50 mM Potassium acetate, 20% glycerol, 1 mM EDTA, 0.01% NP-40 and 5 mm dithiothreitol plus protease inhibitors). Cells were disrupted under liquid nitrogen using an SPEX Certiprep freezer mill (Glen Creston), allowed to thaw on ice, and potassium acetate added to a final concentration of 150 mM before being centrifuged at 90,000 g for 1 h at 4°C. The supernatant was equally divided and applied to protein G beads coupled to the anti-myc antibody 9E10 or with beads alone and rotated for 3 h at 4°C. The beads were washed extensively with extraction buffer containing 500 mM potassium acetate and subsequently washed into a disposable column and the buffer exchanged for TEV buffer (50 mM phosphate buffer pH7.5, 150 mM NaCl, 1 mM b-mercaptoethanol, 10% Glycerol, 0.01% NP-40). The HIS_6_-Smy2 was released from the beads by the addition of TEV protease and incubation overnight on a rotating wheel at 4°C. The supernatants were collected, imidazole added to 10 mM, and incubated with nickel beads for 2 h at 4°C. The beads were washed in TEV buffer containing 10 mM imidazole before elution by boiling for 10 min in SDS-loading buffer. These were loaded onto a 4–12% Bio-rad gel and silver stained or on a 10% Invitrogen gel for short gel lane extraction and mass spectrometry analysis. Mass spectrometry analysis was performed as previously described (Wan et al., 2021).

##### Yeast extracts

Typically, whole cell extracts (WCE) from yeast were prepared by suspending 3 × 10^8^ cells in 750 μL yeast lysis buffer (150 mM Tris-Acetate pH7.4, 100 mM potassium acetate, 1 mM EDTA, 0.1% Triton X-100, 10% glycerol, 1x Protease Inhibitor mix [284ng/mL leupeptin, 1.37 μg/mL pepstatin A, 170ug/mL phenylmethylsulfonyl fluoride and 330ug/mL benzamindine]) in screw cap eppendorf tubes. Approximately 500 μL of 0. 5 mm diameter Glass beads (BioSpec Products) were added, and the cells disrupted using a FastPrep-24 cell homogenizer (MP Biosystems) with 6 rounds of 30 s at 5.5 amplitude. Samples were incubated on ice between disruptions, to reduce heating of the sample. The crude extract was subject to benzonase treatment (20 min, 4°C, 2 units of benzonase/mL of extract) before being clarified twice at 20,000 g for 10 min. Protein concentrations were measured by Bradford assay.

For visualization of pr-Def1 formation *in vivo*, whole cell extracts (WCE) were always prepared by alkaline (denaturing) extraction (Kushnirov, 2000). Briefly, 1–2x10^7^ cells were pelleted and resuspended in 100 mM Sodium Hydroxide for 5 min at room temperature. Cells were pelleted and the supernatant discarded. The pellet was resuspended directly in 1.5x SDS loading buffer and heated to 99°C for 5 min, before placing on ice. Samples were re-heated and spun at 14 000 g for 1 min before loading on SDS-PAGE gels. pr-Def1 is unstable, making it necessary to visualize it by Western blotting immediately after preparation.

To visualise membrane-tethered Spt23, cells were harvested by centrifugation for 3 min at 2,500 g, 4°C and washed in ice-cold PBS before freezing in liquid nitrogen. After defrosting on ice, the pellet was boiled for 3 min at 95°C. 100 μL of 1x SDS loading buffer supplemented with 5 mM N-ethylmaleimide was added, and the microcentrifuge tube was filled half-way with 0.5 mm diameter Glass beads (BioSpec Products). A FastPrep-24 cell homogenizer (MP Biosystems) was used at 6.5 amplitude for 1 min to break open the cells. The supernatant was boiled again for 3 min at 95°C and cleared by centrifugation for 5 min at 16,000 g before loading on SDS-PAGE gels.

##### Co-immunoprecipitation from yeast and MRCV5VA extracts

Immunoprecipitation from yeast cells was carried out on extracts prepared by glass bead lysis. Immunoprecipitation was performed from human cell extracts generated as described below or from combining the Chromatin I and II fractions (described in cell fractionation) to form the Chromatin fraction. Typically, 1 mL extract at 1 mg/mL was incubated with 50 μL beads alone for 1 h at 4°C to pre-clear non-specific binding. GIGYF2 (A303-732) (Bethyl Laboratories Inc. Texas), proteasome (ab109530) (Abcam), HA (12CA5) or myc (9E10) antibodies were pre-incubated with protein A or G beads for 1 h at 4°C before incubation with the pre-cleared extract. M2 anti-flag agarose (Sigma-Aldrich 30410) was used for FLAG tagged purifications. 50 μL of antibody bound beads were rotated with the extracts for 3 h at 4°C. Unbound material was saved and the beads were either washed two times in their immunoprecipitation buffer, followed by once in high salt (500 mM) and then washed back into the applicable immunoprecipitation buffer. Beads were eluted by the addition of 2.5x SDS-loading buffer.

##### Western blot analysis

Typically, 50 μg protein/lane was separated on 4–15% Criterion TGX (BioRad, 5671084) or 3–8% Tris-Acetate Criterion XT (BioRad, 3450130) gels and transferred to nitrocellulose (GE Healthcare Life Sciences, 10600002). Membranes were stained with Ponceau S, scanned and the membranes were blocked in 5% (w/v) skimmed milk in PBS-T (PBS, 0.2% (v/v) Tween 20) for 1 h at room temperature and incubated in primary antibody in 5% (w/v) skimmed milk in PBS-T overnight at 4°C. Primary antibodies are listed in key resources table. For anti-ubiquitin blots, an equal mixture of P4D1 and P4G7 were used. Membranes were washed 3 times in PBS-T, incubated for 1 h at room temperature in HRP-conjugated secondary antibodies (anti-mouse, Santa Cruz, sc516102 or anti-rabbit, Jackson, 711035152) and visualised with SuperSignal™ West Pico PLUS Chemiluminescent Substrate (Thermo Fisher Scientific, 34580). Blots were visualised on Amersham hyperflim ECL or by AI600 Amersham Imager chemiluminescence.

##### Protein purification

Glutathione-S-Transferase (GST), GST-GYF and GST-GAF were expressed as described for the GST affinity resins. After recombinant overexpression, cells were lysed via sonication (30% output 6 cycles 15s on) in GST lysis buffer (PBS, 15 mM Phosphate buffer pH 7.4, 10% glycerol, 0.2% Triton X-100, protease inhibitors, 2 mM β-Mercaptoethanol) containing lysozyme (100 μg/mL). The extract was clarified at high speed and pre-equilibrated glutathione agarose beads (GE healthcare) were added and incubated for 4 h at 4°C.

Cdc48-Flag_3_ was expressed from the endogenous locus of Cdc48. Cells were resuspended in FLAG buffer (150 mM Tris acetate pH 7.8, 150 mM KOAc, 20% glycerol, 0.01% NP40, 1 mM ATP, 1 mM MgCl2, and protease inhibitors) and disrupted under liquid nitrogen using an SPEX Certiprep freezer mill (Glen Creston). After thawing on ice, they were spun for 1 h at 25,000 g to clear the extract. The supernatant was incubated with anti-Flag M2 resin (Sigma-Aldrich) for 2 h at 4°C. Beads were washed in 50 CV Flag buffer and the protein was eluted in Flag buffer + 0.5 mg/mL 3xFlag peptide (synthesised by the peptide synthesis facility at the Francis Crick Institute). Elutions containing protein were pooled together and loaded on a Mono Q column (GE Healthcare). The Mono Q column was run on the AKTA HPLC system with a 20 CV gradient, using Mono Q buffer A (50 mM Tris acetate pH 7.8, 10% glycerol, 1 mM ATP, 1 mM MgCl2) going from 15% to 100% Mono Q buffer B (50 mM Tris acetate pH 7.8, 2M KOAc, 10% glycerol, 1 mM ATP and 1 mM MgCl2). Fractions containing protein were pooled together.

##### Binding assays

Equivalent amounts of GST, GST-GYF and GST-GAF bound beads were incubated with a 10-fold molar excess of pure, recombinant Cdc48-Flag protein, added to the reactions as indicated, in 20 mM Tris pH 7.5, 200 mM NaCl, 0.05% Triton X-100, 1x Protease Inhibitors, 15% glycerol, 75 μg/mL BSA). After a 2-h incubation, beads were washed extensively, re-suspended in SDS-PAGE loading buffer, and analyzed by western blotting. For the reverse binding assay, Cdc48-FLAG was immobilized on Anti-FLAG M2 affinity gel (Sigma-Aldrich) at 2 mg/mL of resin as per manufacturers protocol and binding to GST, GST-GYF and GST-GAF was tested as above.

##### Human cell extract preparation

For preparing whole cell extracts, MRC5VA strains were grown on 15 cm dishes to 80–90% confluency and rinsed twice with ice-cold PBS containing Protease Inhibitors (2.2 mM PMSF, 2 mM Benzamidine HCL, 2 μM Leupeptin, 1 μg/mL Pepstatin A) and 2 mM N-elthylmaleimide (NEM). Cells were then lysed on the plate in TENT lysis buffer (50 mM Tris-HCl pH 7.4, 150 mM NaCl, 2 mM EDTA, 1% (v/v) Triton X-100), containing 2 mM NEM and Protease inhibitors, for 5 min at room temperature (RT). The lysates were collected in 15 mL falcon tubes and kept on ice for 20 min followed by a brief sonication; 10 s at 20% amplitude in a Branson sonicator or Biorupter water bath sonicator (Diagenode) on high 30 s ON/30 s OFF for 7 min. MgCl_2_ to 3 mM and BaseMuncher 1:1000 (Expedeon, BM0100) were added to each sample and incubated for 1 h at 4°C with rotation. Debris was removed by centrifugation 5 min at 20.000 RCF in an Eppendorf microfuge. The extracts were either used directly or snap-frozen in liquid nitrogen.

##### Preparing MultiDsk and GST-Dsk2 affinity resins

Generation of GST-MultiDsk and GST-Dsk2 resin has already been extensively described (Wilson et al., 2012; Tufegdzic Vidakovic et al., 2019). Briefly, One Shot BL21 (DE3) Star bacteria was transformed with pGST-MD or pGEX3-Dsk2 plasmid according to the manufacturer’s instructions. 10 mL of overnight culture was used to inoculate a 250 mL culture grown to OD_600_ = 0.6, all at 37°C in LB with 100 μg/mL Ampicillin (VWR, 171254-25) and shaking. Expression was induced with 1 mM IPTG, and bacteria grown at 30°C with shaking for 4 h, cells were then pelleted and snap frozen in liquid nitrogen.

For GST-MultiDsk resin, cells were lysed, and protein solubilised essentially as described (Frangioni and Neel, 1993). Briefly, thawed pellets were resuspended in STE buffer (10 mM Tris pH 8, 1 mM EDTA, 100 mM NaCl, protease inhibitors) with lysozyme (100 μg/mL). After 15 min on ice N-lauryl sarcosine was added to a final concentration of 1.5%, to denature all proteins. After brief sonication (20% output 4 cycles15s on) and centrifugation at 10 000 g 5 min, Triton X-100 was added to the supernatant to a final concentration of 3%. The Triton (added at a w:w ratio of 1:8) masks the sarcosine by forming mixed micelles (Dekker et al., 2002). Pre-equilibrated glutathione agarose beads (GE healthcare) were added, and the slurry incubated for 2–4 h at 4°C. The beads were washed thoroughly in MultiDsk wash buffer (PBS, 450 mM NaCl, 10% glycerol, 0.1 mM EDTA, 0.1% Triton X-100, 2 mM DTT and protease inhibitors), followed by a low salt wash (50 mM Phosphate buffer pH 7.4, 50 mM NaCl, 10% glycerol, 1 mM β-Mercaptoethanol, 0.2% Triton X-100 and protease inhibitors). The resin was stored as a 4x slurry in PBS +0.01% Sodium azide, at 4°C.

GST-Dsk2 affinity resin was prepared as previously described (Tufegdzic Vidakovic et al., 2019). The pellet was resuspended in PBS with protease inhibitors (2.2 mM PMSF, 2 mM Benzamidine HCL, 2 μM Leupeptin, 1 μg/mL Pep statin A) and sonicated (Branson Digital Sonifer 250) at 30% output for 15 s ON/30 s OFF pulses for a total of 10 min ON time on ice. Triton X-100 was added to 0.5%, mixed gently and incubated on ice for 30 min. Following a 12000 g, 4°C, 10 min spin the supernatant (lysate) was taken and DTT added to 2 mM final concentration. Glutathione Sepharose 4B Beads (Sigma, GE17-0756-01) were spun at 700 g, washed twice in PBS, added to the lysate, and incubated at 4°C with rotation for 4 h. Beads were then spun at 700 g, washed for 5 min twice with ice-cold PBS +0.1% Triton X-100 then once with ice-cold PBS. Final GST-DSK2 affinity resin was resuspended in PBS containing protease inhibitors and 0.02% sodium azide, and stored at 4°C before use.

##### MultiDsk and DSK2 ubiquitin pulldown

MultiDsk pull down was performed on 1 mg of yeast extract prepared by glass bead lysis. The lysate and 15 μL of MultiDsk resin were incubated for 2 h at 4°C before beads were extensively washed with wash buffer (150 mM Tris-Acetate pH 7.4, 500 mM potassium acetate, 1 mM EDTA, 0.1% Triton X-100, 10% glycerol, Protease Inhibitor mix), and then with the same buffer only containing 50 mM potassium acetate. Beads were resuspended in 1.5 times SDS loading buffer and subjected to SDS-PAGE on BioRad Criterion 4–12% or 3–8% Tris-Acetate gels.

For MultiDsk pulldown, one 15 cm dish of 80% confluent MRC5VA-derived cells were used per condition. At indicated times media was removed, cells washed with PBS containing 2 mM NEM (200 mM stock in ethanol made fresh). 800 μL TENT buffer (50 mM TrisHCL pH7.4, 2 mM EDTA, 150 mM NaCl, 1% Triton X-100) containing 2 mM NEM (200 mM stock in ethanol made fresh) and protease inhibitors (2.2 mM PMSF, 2 mM Benzamidine HCL, 2 μM Leupeptin, 1 μg/mL Pep statin A) was added and incubated for 5 min, then scraped into an Eppendorf tubes, collected and snap frozen in liquid nitrogen. Samples were incubated on ice for 20 min then sonicated in a Biorupter water bath sonicator (Diagenode) on high 30 s ON/30 s OFF for 7 min. MgCl_2_ to 3 mM and BaseMuncher 1:1000 (Expedeon, BM0100) were added to each sample and incubated for 1 h at 4°C with rotation. Samples were spun at 20,000 g, 4°C, 5 min and the supernatant taken. Protein concentration was measured (Protein Assay Dye Reagent, BioRad, 5000006) and each sample adjusted with TENT buffer to 750 μL at 1mg/mL, a 1% input was taken and boiled with Sample buffer. 120 μL per sample of bead suspension (30 μL packed bead volume, GST-DSK2 affinity resin) was spun down at 700 g and resuspended in equivalent volume of TENT buffer. 120 μL was added to each sample and rotated overnight at 4°C. Beads were spun down at 700 g, washed at 4°C, rotating incubations twice in TENT buffer and once in PBS for 5 min each. 50 μL 1x Sample Buffer was added and the sample boiled for 2 min 1% input and 20% sample were run on a 4–15% Criterion TGX gel (BioRad, 5671084) and normal Western blotting procedure followed.

##### Proximity ligation assay

Wild type or GIGYF1/2 DKO cells were plated in 8 well slides (PEZGS0816, Sigma). Cells were washed with PBS and fixed for 20 min with 3.7% formaldehyde, washed twice in PBS and permeabilised for 5 min with PBS + 0.2% Triton and washed in PBS. Proximity Ligation Assay (PLA) was carried out using Duolink Kit (DUO92101) from Sigma following manufacturer instructions, except PLA probes were diluted 1:10. Antibodies used were: p97 (ab11433, Abcam) 1:300, GYGF2 (24790-1-AP, Proteintech) 1:300, UFD1 (10615-1-AP, Proteintech) 1:100. Images were obtained with a Leica SP5.

##### Cellular fractionation

Cells were plated in 15 cm plates to be around 80% confluent the following day when they were UV irradiated at 20 J/m^2^. At time points indicated cells were washed with ice-cold PBS, scraped into an Eppendorf tube in ice-cold PBS and pelleted at 300 g, 4°C, 5 min. Cellular fractionation was carried out as previously described (Gregersen et al., 2019). All buffers contained cOmplete protease inhibitor (Sigma, 5056489001), phosSTOP phosphatase inhibitor (Sigma, 4906837001) and 2 mM NEM. Pellets were resuspended in 500 μL hypotonic buffer (10 mM HEPES pH 7.5, 10 mM KCl, 1.5 mM MgCl_2_) and incubated on ice for 15 min, homogenised with 20 strokes using a loose pestle and spun at 3000 g, 4°C, 15 min. Supernatant was taken as the cytoplasmic extract and corrected to 10% (v/v) glycerol, 3 mM EDTA, 0.05% (v/v) NP-40, 150 mM NaCl. The remaining nuclear pellets were resuspended in 500 μL nucleoplasmic extraction buffer (20 mM HEPES pH 7.5, 1.5 mM MgCl_2_, 150 mM Potassium Acetate, 10% v/v Glycerol, 0,05% (v/v) NP-40) and incubated on ice for 20 min, then spun at 20,000 g, 4°C, 20 min to pellet chromatin. Supernatent was taken as the Nucleoplasmic fraction. The remaining chromatin pellets were resuspended in 200 μL chromatin digestion buffer (20 mM HEPES pH 7.9, 1.5 mM MgCl_2_, 150 mM NaCl, 10% (v/v) Glycerol, 0.05% (v/v) NP-40, 1:1000 BaseMuncher (Abcam, ab270049)) and incubated for 1 h, 4°C, rotating, then spun at 20,000 g, 4°C, 20 min, supernatant was taken as the low salt chromatin fraction (Chromatin I). Pellets were resuspended in 120 μL high salt chromatin extraction buffer (20 mM HEPES pH 7.9, 500 mM NaCl, 3 mM EDTA, 1.5 mM MgCl_2_, 10% v/v Glycerol, 0,05% (v/v) NP-40) and incubated on ice for 20 min 280 μL high salt dilution buffer (20 mM HEPES pH 7.9, 3 mM EDTA, 1.5 mM MgCl_2_, 10% (v/v) Glycerol, 0,05% (v/v) NP-40) was added and samples spun at 20,000 g, 4°C, 15 min. Supernatent was collected as the high salt chromatin fraction (Chromatin II). Protein concentration was measured (Protein Assay Dye Reagent, BioRad, 5000006) in a spectrophotometer or a plate reader.

##### Incucyte apoptosis analysis

2 × 10^3^ cells/well were plated in black 96 well plates (Greiner Bio-one 655090) and treated with either DMSO or 10uM NMS873 (Tebu-Bio Ltd.) and 4uM NucView 488 Caspase-3 Assay Kit (Biotium, California). Fluorescence data were collected in an Incucyte Live Cell Analysis System (Essen Bioscience) over 15 h and data analyzed with Graphpad Prism 9.

##### Cell images after NMS873 treatment

For visualisation of cells, 5 × 10^3^ cells were seeded and treated as above. Images of WT and GIGYF1/2 KO cells showing Phase and Green after 12 h in the Incucyte were taken.

##### Caspase 3/7 luminescence assay

5 × 10^3^ MRC5VA or GIGYF1/2 knock-out cells were seeded in a 96-well, white, clear bottom assay plate (Corning, Costar 3610). Next day, an equal volume of 20uM NMS873 (Tebu-Bio Ltd.) 2uM CB-5083 (Cambridge Bioscience) or DMSO was added to the wells and incubated for 18 h. An equal volume of Caspase-Glo 3/7 assay reagent (Promega Corporation) was added to each well, according to the manufacturer’s instructions. Luminescence was measured in a PHERAstar PlateReader (BMG Labtech).

### Quantification and statistical analysis

Statistical details of experiments can be found in the figure legends. For proximity Ligation Assays, maximum projection images were processed in Fiji - the DAPI channel was used to segment nuclei with a Median filter radius 2, Otsu thresholding, Fill holes, Watershed, then particles selected with a minimum area of 15. Nuclei were processed with Voronoi segmentation to estimate cell boundaries followed by Percentile threshold then particles selected with a minimum area of 15. Segmentation was manually checked and corrected or excluded when necessary, including excluding partial cells. PLA signals were processed with Find Maxima with prominence 20 and the number per estimated cell area measured. Statistics were calculated in Prism using One-way ANOVA followed by Dunnett’s multiple comparisons comparing all conditions to Wild type with p97 and GIGYF2 antibodies. Statistical analysis was carried out in Prism 7 software, no methods were used to determine if data met assumptions of the statistical approach. Representative experiment of 2 repeats shown. For Incucyte quantification, the results were analyzed using GraphPad Prism 9 Software and statistical analysis was by the unpaired t test.

## Data Availability

• There are no large-scale datasets in this manuscript. • The manuscript does not report original code. • Any additional information required to reanalyze the data reported in this paper is available from the lead contact upon request.

## Supplemental information

Document S1. Figures S1–S3 and Table S2

Table S1. Smy2 interactome, related to Figure 2

Document S2. Article plus supplemental information
