# Supplementary material for: Yeast Smy2 and its human homologs GIGYF1 and -2 regulate Cdc48/VCP function during transcription stress
Source: Cell Rep. 2022 Oct 25;41(4):111536. doi: 10.1016/j.celrep.2022.111536 (PMC9638028; doi:10.1016/j.celrep.2022.111536)
Supplement: Document S1. Figures S1–S3 and Table S2 [file mmc1.pdf]

Cell Reports, Volume *41*

Supplemental information

Yeast Smy2 and its human homologs GIGYF1 and -2 regulate Cdc48/VCP function during transcription stress

Michelle Harreman Lehner, Jane Walker, Kotryna Temcinaite, Anna Herlihy, Michael Taschner, Adam C. Berger, Anita H. Corbett, A. Barbara Dirac Svejstrup, and Jesper Q. Svejstrup

Supplemental Figures with Legends

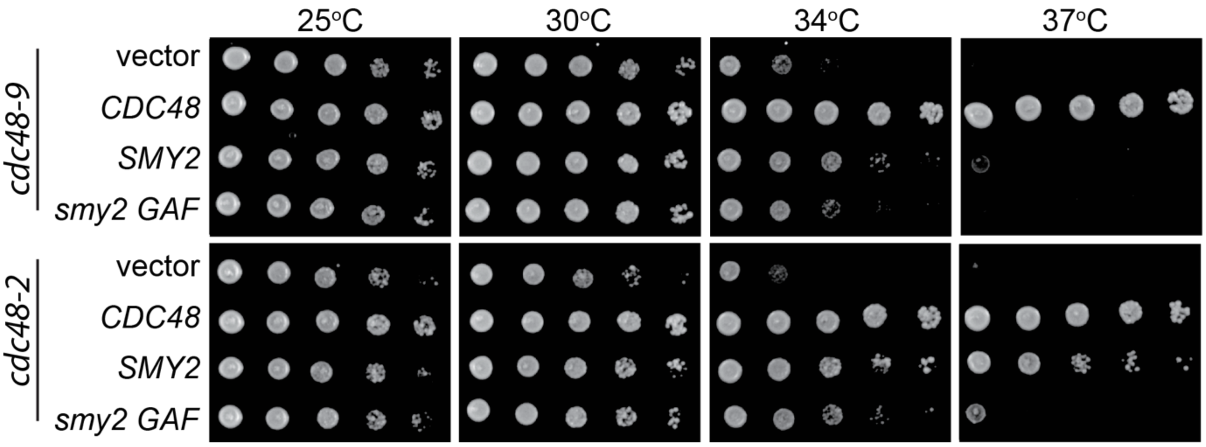

Supplementary Figure S1, related to Figure 2. SMY2 also affect growth of other *cdc48* mutants. Dilution series of *cdc48-9* and *cdc48-2* yeast cells (carrying the indicated *SMY2* or *CDC48* plasmids) plated on minimal media and grown at the stated temperatures for 2-4 days.

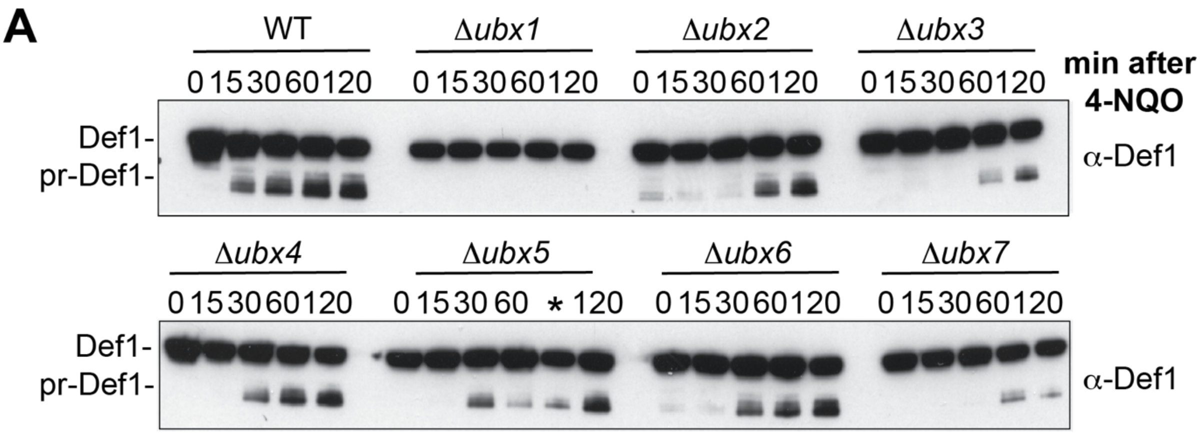

Supplementary Figure S2, related to Figure 5. Effects of *UBX* genes and *SMY2.* A. Western blot analysis of Def1 from extracts of wild type and deletion strains of the seven yeast Ubxs. Logarithmically growing cells were incubated with 4-NQO for the indicated times. *Denotes a gel loading error. There is variability in Def1 processing assays, but only *ubx1* deletion consistently had a dramatic effect in such experiments.

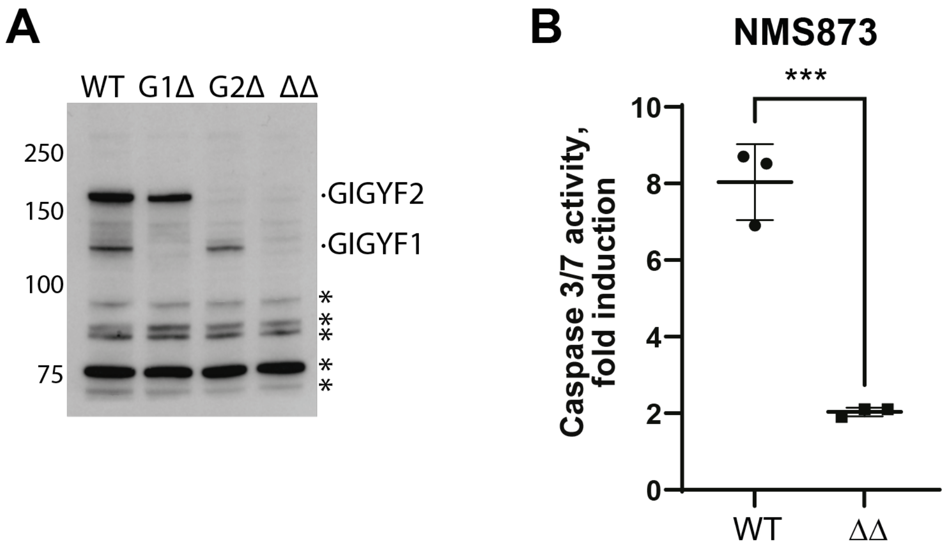

Supplementary Figure S3, related to Figures 6 and 7. A *GIGYF1/2* knockout and its effect on p97/VCP inhibitor. A. Step-wise generation of *GIGYF1* and *GIGYF2* double knockout cells. **B.** The apoptosis-inducing effect of p97/VCP inhibitor NMS873 and the dependence on the GIGYF proteins, 15 hours after adding inhibitor. Only clone C12 tested. Data from 3 biological replicates; data is average of 3 independent wells in each condition in each experiment. ***P-value <0.001. Results were analysed using GraphPad Prism 9 Software and statistical analysis by the unpaired t-test showed a p-value of 0.0005.

Supplementary Table S2. Oligonucleotides. Related to STAR Methods and Key Resources Table.

REAGENT OR RESOURCE	SOURCE	IDENTIFIER
Forward SMY2	CCAATATCTACCGCCAGTGATGC	N/A
Reverse SMY2	GGGTAGACGCATCTTATTACCCGC	N/A
Forward Smy2 deletion	CCTTGAGCTTTTACCTTCCTTCCTCCTCCCCTATATACTC<br>AACTTCTCAGCCCACATCAATATCCGGTTCTGCTGCTAG<br>T	N/A
Reverse Smy2 deletion	GTATATACAATAACAATAAATGATAAAGAAATATGCAG<br>TGAAAAGAAAAAATTATGAAGCTTTTCCTTCCTCGAGGC<br>CAG<br>AAGAC	N/A
Forward FLAG-Smy2	CGGATCCACTAGTAACGGCCGCCAATGGATTACAAGGA<br>TGACGACGATAAGGGCGGAATAGCACCAGACTCGCAAA<br>GATT<br>ATTCG	N/A
Reverse FLAG-Smy2	TCCGCCCTTATCGTCGTCATCCTTGTAATCCATTGGCGG<br>CCGTTACTAGTGGATCCGAGCTCG	N/A
Forward Smy2 GAF mutant	CATAGGTGGCGCTTTTGCTTCAACC	N/A
Reverse Smy2 GAF mutant	TACCATTGTGACATCATTTG	N/A
Forward GST-Smy2 GYF domain	GCAGGGCTGGCAAGCCACGTTTGGTGG	N/A
Reverse GST-Smy2 GYF domain	GACCGTCTCCGGGAGCTGCATGTGTCAGAGG	N/A
Forward GST-Smy2 GYF domain GAF mutant	GGTCCATTTACTACCCAAATGATGTCACAATGGTACATA<br>GGTGGCGCTTTTGCTTCAACCC	N/A
Reverse GST-Smy2 GYF domain GAF mutant	CCCAATCTTGAAATCTGAAGGGTTGAAGCAAAAGCGCC<br>ACCTATGTACCATTGTGACATCATTTGG	N/A
Forward FLAG-Cdc48 integration	CCACTAGCTAAAAAGTGGAACGATCATTCAAGAGATCC<br>CCGGTTATATGCCAGGTATATTTTTATTTTAAATCGTAA<br>ATT<br>CAAGTC	N/A
Reverse FLAG-Cdc48 integration	GACTTGAATTTACGATTTAAAATAAAAATATACCTGGCA<br>TATAACCGGGGATCTCTTGAATGATCGTTCCACTTTTTA<br>GC<br>TAGTGG	N/A
Forward Cdc48	CTGGCAAGCTTGAAGTAAAAGGACAATCAGCACGCCTT<br>CC	N/A
Reverse Cdc48	CGGATCTCGAGCCAATAACATTAGCGACAAGTTTTCTCC<br>GCG	N/A
Forward Pre1-myc	GGGCGTCATTGTTAAAATCGTGGATAAAGATGGCATAA<br>GACAAGTAGATGACTTCCAGGCACAGTCCGGTTCTGCT<br>GCTA<br>GT	N/A
Reverse Pre1-myc	GGAAGATAATTACTTTAGTATATCATTAGCAATCACCTT<br>TTCCGTGTGATTACACTGAATATCTTTCACCTCGAGGCC<br>AG<br>AAGAC	N/A

Forward FLAG-Spt23	GATTATAAAGATGACGATGACAAGATGATGAGTGGCACAGGAAAC	N/A
Reverse FLAG-Spt23	CTTGTCATCGTCATCTTTATAATCCTTATCGTCGTCATCCTTG	N/A
GIGYF1 gRNA	TGACTACCGTTATGGGCGAG	N/A
Forward GIGYF1	ACTCGAGCTTCCCATCTCCT	N/A
Reverse GIGYF1	CCGAAATAAGCACCCCCAGA	N/A
GIGYF1 Sequencing	GGCGGGAGTGAGGACCCAGGC	N/A
GIGYF2 gRNA	ATTTCTGCCTATCCTCCAGG	N/A
Forward GIGYF2	TCACTTGAGAAGCTGGGGAGT	N/A
Reverse GIGYF2	AGGATGGTTCCCAATGTCCTT	N/A
GIGYF2 Sequencing	CTGGGGAGTATTGACTGGGGT3	N/A
